# Biocompatible Ink Optimization Enables Functional Volumetric Bioprinting With Xolography

**DOI:** 10.1002/adma.202512058

**Published:** 2025-11-29

**Authors:** Erik Brauer, Aiste Balciunaite, Matthias R. Kollert, Julian Weihs, Raphael S. Knecht, Rose Behncke, Susanna Quach, Niklas Felix König, Asia Badolato, Stella Monestier, Simone Bersini, Matteo Moretti, Miriam Filippi, René Hägerling, Milad Rezvani, Stefan Hecht, Ansgar Petersen, Robert K. Katzschmann

**Affiliations:** ^1^ Center for the Science of Materials Berlin (CSMB) and Department of Chemistry Humboldt University 12489 Berlin Germany; ^2^ Soft Robotics Laboratory Department of Mechanical and Process Engineering ETH Zurich Tannenstrasse 3 Zurich 8092 Switzerland; ^3^ Julius Wolff Institute – Center for Musculoskeletal Biomechanics and Regeneration Berlin Institute of Health at Charité – Universitätsmedizin Berlin 13353 Berlin Germany; ^4^ BIH Center for Regenerative Therapies Berlin Institute of Health at Charité – Universitätsmedizin Berlin 13353 Berlin Germany; ^5^ Department of Pediatrics Division of Gastroenterology Nephrology and Metabolic Medicine Charité – Universitätsmedizin Berlin 13353 Berlin Germany; ^6^ xolo GmbH 12489 Berlin Germany; ^7^ Regenerative Medicine Division Institute for Translational Research (IRT) Faculty of Biomedical Sciences Università della Svizzera italiana and Ente Ospedaliero Cantonale Via Chiesa 5 6900 Bellinzona Switzerland; ^8^ Euler Institute, Faculty of Biomedical Sciences Università della Svizzera italiana Via Buffi 13 Lugano 6900 Switzerland; ^9^ Servizio di Ortopedia e Traumatologia Ente Ospedaliero Cantonale Via Tesserete 46 Lugano 6900 Switzerland; ^10^ ETH AI Center ETH Zurich Andreasstrasse 5 Zurich 8092 Switzerland; ^11^ ETH RobotX ETH Zurich Leonhardstrasse 21 Zurich 8092 Switzerland

**Keywords:** 3D printing, biohybrid robotics, bioprinting, tissue engineering, Xolography

## Abstract

Xolography is a novel linear volumetric manufacturing technique that offers unparalleled precision and speed. Yet, its application to bioprinting remains limited due to insufficient understanding of biocompatibility constraints. Here, this work establishes fundamental design principles for cell‐compatible Xolography bioinks by dissecting the effects of extracellular pH, osmolality, and lysosomotropic stress on cell viability and function. By systematically studying the tolerances for these parameters, this work defines a framework for bioink formulations that enables fast, support‐free fabrication of complex designs with maintained cell viability and function as validated in different murine and human cell lines, primary human cells and induced pluripotent stem cell (iPSC)‐derived cells. These results show that, unlike triethanolamine, BisTris indeed can function as a fully biocompatible co‐initiator enabling cell viability beyond 90% as well as uncompromised metabolic activity and differentiation performance when used in a tightly controlled formulation, contrasting previous reports. This work showcases the biomedical potential of the formulation by achieving fibroblast‐driven extracellular matrix (ECM) formation, endothelial sprouting from pre‐vascularized spheroids, and maintenance of an iPSC‐derived hepatocyte differentiation phenotype within Xolography‐printed constructs. These advancements transform Xolography into a powerful and foremost reliable bioprinting platform for fabrication of complex, cell‐laden structures for versatile applications in tissue engineering, organ‐on‐a‐chip models, and regenerative medicine.

## Graphical abstract

Xolography's reliance on weak‐base co‐initiators introduces unique biochemical constraints. By dissecting the effects of extracellular pH, osmolality, and lysosomotropic stress, this study defines the biochemical design space that enables fully biocompatible, cell‐laden volumetric printing.

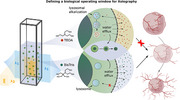

## 1 Introduction

Biofabrication of cell‐laden constructs aims at replicating the hierarchical complexity of native tissues in vitro to produce tissue and disease models, organ‐on‐a‐chip systems and eventually grafts for implantation.^[^ ^1^ ^]^ To achieve the complexity of tissues, printed structures need to display high fidelity at the microscale (≈10 µm) and the macroscale (≈300 µm) in order to drive hierarchical cell and tissue self‐assembly – for example when engineering vascular beds.^[^ ^2^ ^]^ Engineering such hierarchically structured constructs hereby requires advanced additive manufacturing techniques.

While extrusion‐based and digital light processing (DLP) bioprinting can fabricate centimeter‐scale, multi‐material tissues and achieve high lateral resolution, they remain constrained in freedom of design, fabrication speed, and geometric fidelity compared to volumetric approaches.^[^ ^3^, ^4^, ^5^, ^6^, ^7^ ^]^ Other techniques such as two‐photon polymerization achieve sub‐micron resolution with high fidelity and flexibility, but are limited in the achievable volume generation rate, that is, print speed.^[^ ^8^, ^9^ ^]^ In contrast, light‐based volumetric additive manufacturing offers high‐resolution and scalable printing by leveraging optical projections. One such approach, computed axial lithography (CAL), achieves rapid printing of centimeter‐scale hydrogel constructs with resolutions of 40–100 µm.^[^ ^10^ ^]^ Based on the successive overlay of calculated light patterns from different angles, a light dose distribution is achieved within the volume and above‐threshold dosage results in material hardening. However, polymerization can become non‐uniform as both the photoinitiator and cured material absorb or scatter light. This results in intrinsic polymerization gradients in the hydrogel constructs that affect cell behavior. Additionally, standalone microstructures remain limited to ≈500 µm, restricting the ability to precisely mimic native tissue microarchitecture.^[^ ^11^ ^]^

A recent advancement in light‐based biofabrication is Filamented Light (FLight), which uses an optical modulation instability to create light beams for patterned polymerization that allow fabrication of microstructures in the range of 2–30 µm.^[^ ^12^ ^]^ These patterns have been shown to drive cell alignment, where sequential exposures can generate multi‐directional architectures.^[^ ^13^ ^]^ However, each exposure only creates structures along a single axis, requiring multiple steps for 3D complexity. Additionally, filament penetration is typically restricted to a few millimeters due to scattering and absorption—constraints that become more pronounced at biologically relevant cell densities.

To overcome the non‐uniform polymerization of CAL and FLight, Xolography has emerged as a novel volumetric additive manufacturing technique that enables precise 3D feature control. Unlike conventional photoinitiator systems, Xolography employs a dual‐color photoinitiation chemistry, where UV light induces photoisomerization of a dormant photoinitiator (spiropyran) to an active form (merocyanine), which can trigger radical polymerization selectively by an orthogonal visible light stimulus.^[^ ^14^ ^]^ This approach confines radical formation locally to the intersection volume of a UV and a visible light beam, improving spatial precision and eliminating the shielding effects seen in CAL. Recent work has adapted Xolography for hydrogel‐based printing, achieving a resolution down to 20 µm and demonstrating tuning of local material stiffness through visible light intensity modulation.^[^ ^15^, ^16^ ^]^ However, these studies primarily established technical feasibility and focused on photochemistry, voxel control, and printing performance through supplementation with chemical enhancers, while the biological compatibility of the employed bioinks remained largely unexplored. To date, no study has defined the biochemical design space of Xolography across multiple, biomedically relevant cell types. A clear necessity for a better understanding of biocompatibility aspects arises from the observation that Gelatin Methacryloyl (GelMA)‐based formulations used in Xolography so far have shown biocompatibility issues despite being a widely accepted biocompatible hydrogel.^[^ ^15^ ^]^ Xolography depends on weak tertiary amines as co‐initiators for starting radical formation which themselves potentially act as biological stressors. Thus, design rules from other bioprinting setups cannot simply be adopted and a fully biocompatible protocol and description of cell functionality is needed to overcome these issues and facilitate the broad adoption of this technology.

Here, we establish a biochemical framework that defines parameters for fully biocompatible, cell‐laden bioprinting with Xolography. We identified alkaline pH, lysosome alkalization and medium tonicity influenced through intrinsic physicochemical properties of the co‐initiator as extracellularly and/or intracellularly acting stressors of cell viability. By replacing triethanolamine (TEOA) with BisTris in combination with an optimized nutrient supplementation, we achieved >90% cell viability across various cell types in GelMA‐printed constructs. This defines the biological “safe operating space” for Xolography and enables future applications to build on a robust and reproducible biochemical foundation. We further validated our approach by printing primary cells, iPSC‐derived cells, and multi‐cellular aggregates, assessing tissue formation, metabolic activity, and vascular network development. Our findings demonstrate the feasibility of Xolography for bioprinting, offering a scalable approach for engineering cell‐laden constructs with high precision. This advancement brings Xolography closer to applications in organoid research, personalized medicine, and tissue engineering.

## 2 Results

### 2.1 Requirements for a Biocompatible Printing System for Xolography

**Figure 1:**
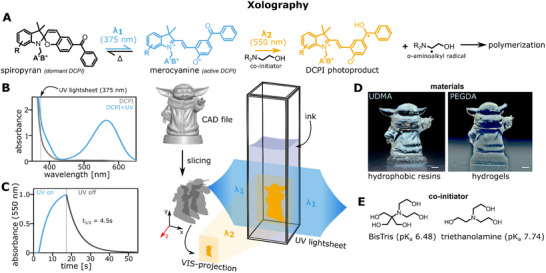
A dual‐color photoiniator (DCPI) enables local photopolymerization by orthogonally intersecting light sources. A) **S**chematic of the DCPI's photoisomerization upon light activation and subsequent radical formation at α‐position of a tertiary amine. A^−^B^+^ denotes an ionic group to allow water solubility. R denotes an organic group as a substituent at any given free position. B) Absorbance spectrum of the DCPI either in the dark (gray line) or during irradiation (blue line) with a 375 nm UV light source (gray dashed line indicating light source). C) Absorbance at 550 nm after irradiation for 15 s (blue) and observation of thermal decay of the active merocyanine to the dormant spiropyran (gray). The half‐life of 4.5 s was calculated from an exponential decay fit of the absorbance signal after switching off the UV light source. D) Test prints demonstrating the process‐intrinsic capacity of Xolography using either urethane‐based hydrophobic resin (xoloOne, denoted “UDMA”) or a PEGDA‐based hydrogel formulation (xoloPEGDA, denoted “PEGDA”). Scale bar 1 mm. E) Chemical structures of selected co‐initiators, BisTris and triethanolamine.

The dual‐color photoinitiator (DCPI) is of type II, that is, the required starting radicals for polymerization are generated by a redox reaction at a co‐initiator molecule. Here, the latter is a tertiary amine that reacts in a fast electron transfer with the excited state of the active DCPI in its merocyanine form (**Figure** **1**A).^[^ ^17^ ^]^ A modified DCPI was used to improve water solubility and photochemical performance under physiological pH conditions. Upon UV irradiation (375 nm), the dormant DCPI can isomerize to the active DCPI. The latter exhibited characteristic visible light absorption with an absorbance peak at 550 nm as well as a thermal relaxation to the initial dormant DCPI isomer with a half‐life of 4.5 s (Figure 1B,C). We furthermore observed stable photochemical performance with only mild differences in relaxation speed between pH 6 to 9 which only declined in a more acidic environment of pH 5 to 4 which is out of scope for bioprinting applications (Supplementary Figure S1A–C, Supporting Information). This makes the new photoinitiator compatible both with synthetic, Polyethylene Glycol Diacrylate (PEGDA)‐based hydrogel formulations achieving similar resolution as with non‐aqueous resins (Figure 1D) but also GelMA‐based biocompatible formulations requiring a physiological pH as presented recently.^[^ ^15^ ^]^

**Figure 2:**
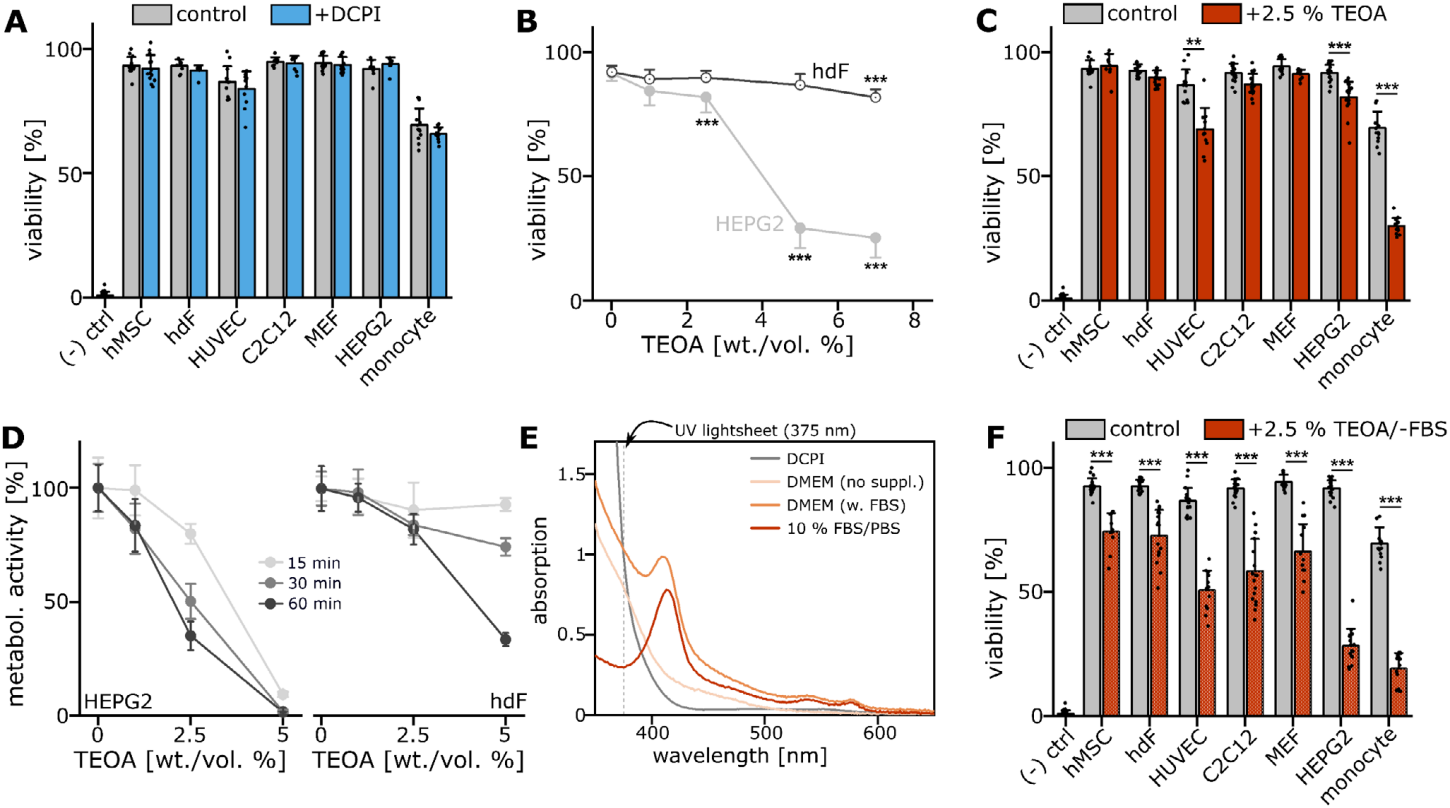
Triethanolamine displays cytotoxic effect across many cell types. A) Viability (% of live cells) of hMSCs, hdFs, HUVECs, C2C12, MEFs, HEPG2 and primary human monocytes after 60 min of exposure to 0.24 mg mL^−1^ DCPI dissolved in the respective expansion medium. A 0.1% Saponin sample served as negative control for reference. N ≥ 8 from at least two independent experiments. B) Viability (% of live cells) of HEPG2 and hdF cells shown as a function of TEOA concentration ranging from 1 to 10% (wt. %) after 60 min of exposure. N ≥ 8 from at least two independent experiments. C) Viability (% of live cells) of hMSCs, hdFs, HUVECs, C2C12, MEFs, HEPG2 and primary human monocytes after 60 min of exposure to 2.5 wt./vol. % TEOA diluted in the respective expansion medium. N ≥ 8 from at least two independent experiments. D) Metabolic activity (normalized to expansion medium control) of HEPG2 (left) and hdF (right) cells exposed to varying concentrations of TEOA for 15, 30 or 60 min. N ≥ 8 from at least two independent experiments. E) Absorbance spectra of the DCPI (gray), DMEM basal medium (light orange), DMEM supplemented with 10% FBS (orange) and 10% FBS diluted in PBS (dark orange). F) Viability (% of live cells) of hMSCs, hdFs, HUVECs, C2C12, MEFs, HEPG2 and primary human monocytes after 60 min of exposure to 2.5 wt./vol. % TEOA dissolved in the respective basal medium (FBS‐free). N ≥ 8 from at least two independent experiments. Statistics via a two‐sided Mann‐Whitney‐U test. A value of <0.05 was considered as statistically significant. Individual significance levels are indicated as follows: *: *p* < 0.05, **: *p* < 0.01, ***: *p* < 0.001.

Due to the high toxicity of low molecular weight acrylates (e.g., PEGDA), a viable biofabrication setup requires non‐toxic, crosslinkable polymers such as GelMA. In addition, ink formulations in Xolography require a tertiary amine acting as co‐initiator next to the DCPI molecule itself. Aside from TEOA with a pK_a_ above physiological pH (pK_a_ 7.74), also BisTris (pK_a_ 6.48) has been explored as a suitable co‐initiator for Xolography (Figure 1E).^[^ ^18^, ^19^ ^]^ However, for single cells in suspension a strongly reduced metabolic activity (25% residual activity) was observed when exposed to high concentrations of BisTris in a particular formulation.^[^ ^15^ ^]^ We started by testing the toxicity of the modified DCPI. We did not detect any signs of imminent or chronic toxicity for a variety of different cells including human Mesenchymal Stromal Cells (hMSC), human dermal Fibroblasts (hdF), human Umbilical Vein Endothelial Cells (HUVEC), mouse myoblast C2C12 and mouse embryonic fibroblast (MEF) cells lines, human HEPG2 and human primary monocytes (**Figure** **2**A) and in presence of multiples of the required DCPI concentration (Supplementary Figure S2A–C, Supporting Information).

Next, we analyzed the toxicity of the co‐initiator TEOA that is required at concentrations of at least 2 wt./vol. % to allow successful printing.^[^ ^20^ ^]^ We initially tested the effect of TEOA on the viability of HEPG2 and hdF cells for concentrations of 1 to 7.5%. While HEPG2 showed a pronounced reduction in viability already at concentrations above 2.5%, hdFs remained viable even at high concentrations and even after 60 min of exposure (Figure 2B). Since 2.5% represented a critical concentration, we compared viability of all cell types at this TEOA concentration (Figure 2C) and observed a reduced viability for HEPG2, HUVEC and particularly for primary monocytes compared to controls. This negative effect was further visible in the metabolic activity 24 h after exposure, when a significant reduction was observed for all analyzed cell types (Supplementary Figure S2D, Supporting Information). As the chosen exposure time of 60 min reflected a multiple of actual printing times of few minutes, we analyzed how the time of incubation affected the toxicity. We observed that the metabolic activity (24 h after treatment) decreased in a time‐dependent manner yet even a relatively short exposure time of 15 min with 2.5% TEOA reduced the metabolic activity of HEPG2s, whereas hdFs were only mildly affected (Figure 2D).

The data shown so far did not reflect actual printing conditions as for the viability testing the examined components were diluted in respective cell expansion media which contain various nutrients and supplements that were so far not included in ink formulations for Xolography. Thus, we analyzed the absorbance of basal cell culture media and of fetal bovine serum (FBS), which can be considered as the most relevant supplement for viability and observed high absorbance at the wavelength of the UV light sheet for FBS‐containing media (Figure 2E). The presence of FBS resulted in a higher optical density that would lead to inhomogeneous light intensity distribution profiles across the build volume.^[^ ^14^ ^]^ Therefore, we concluded that FBS should not be added during a printing process. Consequently, we tested how removal of FBS from our setup affects cellular viability. All analyzed cell types showed a significantly reduced viability and metabolic activity which highlights the protective function of FBS in this context (Figure 2F, Supplementary Figure S2F, Supporting Information).

With regard to the photoinitiation system, the DCPI itself showed no signs of toxicity. However, the co‐initiator TEOA exhibited very limited biocompatibility even for short exposure times which was exacerbated in a FBS‐deficient environment.

### 2.2 Medium Tonicity and pH Compromise Cell Viability

**Figure 3:**
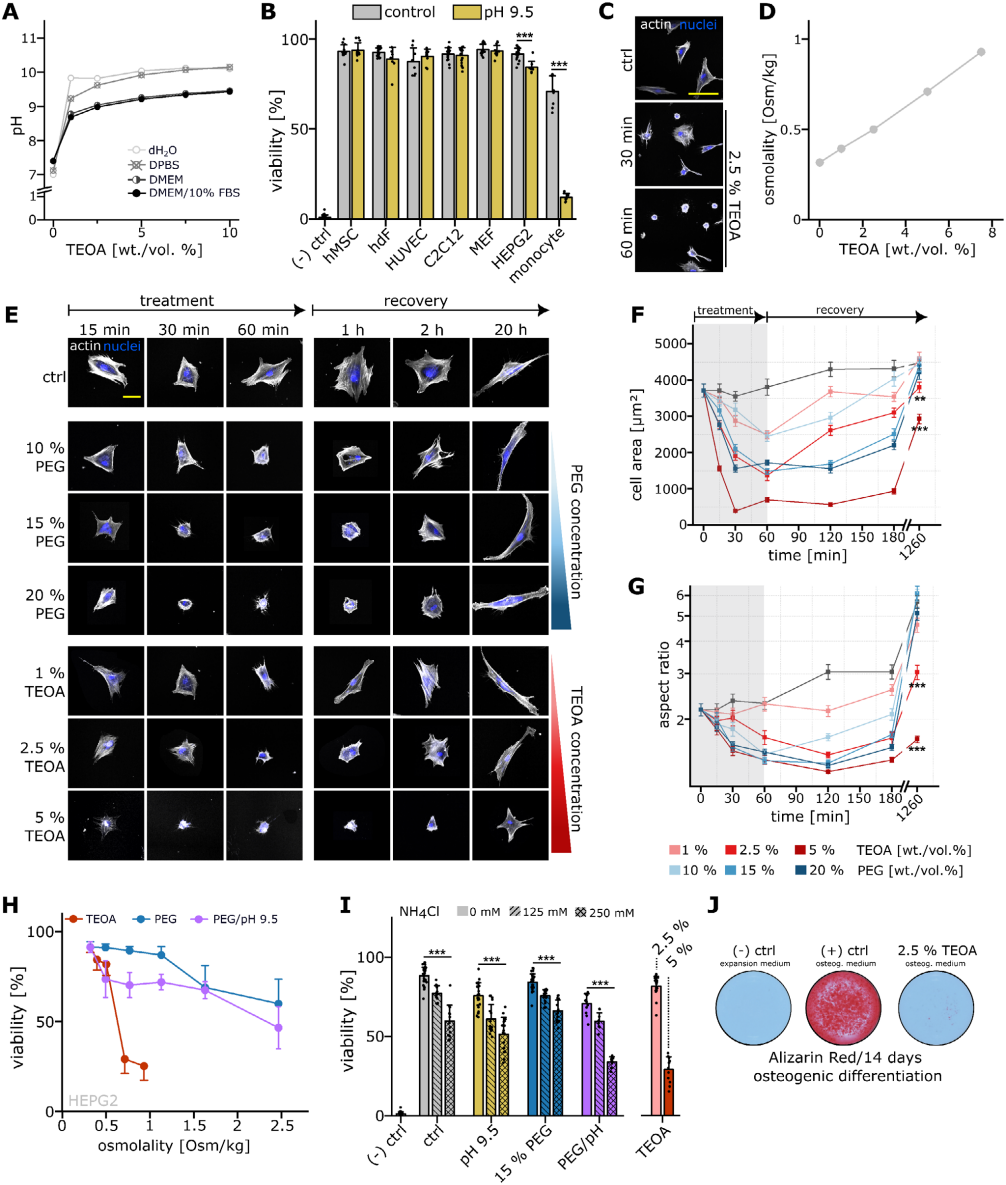
Triethanolamine affects cell viability through alkaline pH, lysosome alkalization and osmotic pressure. A) Measurement of pH as a function of TEOA ranging from 1% to 10%. (wt./vol.) TEOA was either diluted in pure water, PBS and DMEM with or without 10% (vol./vol.) FBS supplementation. B) Viability (% of live cells) of hMSCs, hdFs, HUVECs, C2C12, MEFs, HEPG2 and primary human monocytes after 60 min of exposure to pH 9.5 expansion medium. A 0.1% Saponin sample served as negative control for reference. N ≥ 8 from at least two independent experiments. C) Confocal Images of fibroblasts exposed to 2.5% TEOA diluted in expansion medium for 30 or 60 min. Cells were stained for nuclei (blue) and cytoskeleton (actin, white). Scale bar 100 µm. D) Medium osmolality measured by freezing point depression of a TEOA solution diluted in growth medium (1% ‐ 7.5%, wt./vol.). E) Representative confocal images of hdFs either mock treated (ctrl) or a varying concentration of PEG (molecular weight: 400 g mol^−1^) or TEOA diluted in growth medium for a maximum amount of 60 min. After 60 min, the treatment medium was replaced by fresh expansion medium and the morphology was followed for a maximum of 20 h to observe morphological recovery. Scale bar 50 µm. F) Quantification of cell area as a function of time for TEOA (light to dark red) or PEG (light to dark blue)‐containing media of varying concentration. A mock‐treated sample served as control (dark gray). The light gray box indicates the treatment time window after which the treatment medium was replaced by fresh growth medium. N ≥ 50 cells of at least two independent experiments. Data are depicted as mean ± SEM. G) Quantification of cellular aspect ratio as a function of time for TEOA (light to dark red) or PEG (light to dark blue)‐containing media of varying concentration. A mock‐treated sample served as control (dark gray). The light gray box indicates the treatment time window after which the treatment medium was replaced by fresh growth medium. N ≥ 50 cells of at least two independent experiments. Data are depicted as mean ± SEM. H) Viability (% of live cells) of HEPG2 cells shown as a function of medium osmolality for either TEOA (1% ‐ 7.5% diluted in growth medium, red line), PEG (5% ‐ 25% diluted in growth medium, blue line) or PEG titrated to pH 9.5 (5% ‐ 25% diluted in growth medium, purple line). N ≥ 8 from at least two independent experiments. I) Viability (% of live cells) of HepG2 as a function of NH_4_Cl concentration (0 mM: blank, 125 mM: diagonally striped, 250 mM: diagonal cross) for controls (gray), alkaline pH (pH 9.5, yellow), osmotic pressure (15% PEG, blue) or a combination of both (15% PEG + pH 9.5, purple). TEOA (red) served as a reference for viability. A 0.1% Saponin sample served as negative control for reference. N ≥ 10 from at least three independent experiments J) Representative Alizarin Red stains after 14 days of osteogenic differentiation of hMSCs which were treated for 60 min with 2.5% TEOA diluted in FBS‐containing growth medium. Statistics via a two‐sided Mann‐Whitney‐U test. A value of <0.05 was considered as statistically significant. Individual significance levels are indicated as follows: *: *p* < 0.05, **: *p* < 0.01, ***: *p* < 0.001.

In order to optimize cell viability in Xolography, we aimed to understand the reason for the cytotoxic effects of TEOA. A key feature of TEOA as a co‐initiator is its basicity, that is, pK_a_ = 7.74, which exceeds physiological pH. According to the Henderson‐Hasselbalch equation, at pH 7.4, 68.6% of TEOA molecules are protonated and thus cannot serve as co‐initiator in the photoredox process, which would require higher absolute co‐initiator concentrations to maintain reactivity (Supplementary Figure S3A, Supporting Information). Therefore, we measured pH‐changes of a non‐adjusted TEOA solution where little protonation is expected and observed that a concentration of 2.5% raised the pH above 9 for all conditions (**Figure** **3**A). For higher TEOA concentrations, the pH was not increasing significantly, peaking at 9.5 for DMEM containing 10% TEOA. We therefore continued to measure the viability in response to pH 9.5 as this was the average value for all groups containing TEOA between 2.5 and 7.5%. However, apart from highly pH‐sensitive monocytes, pH 9.5 did not significantly impact viability and metabolic activity, suggesting that alkalinity alone does not fully account for TEOA's cytotoxicity (Figure 3B, Supplementary Figure S3B, Supporting Information). Furthermore, HUVECs exhibited morphological changes due to the differences in pH, which points to limited biocompatibility of an alkaline printing environment when it comes to working with more sensitive cells such as hepatocytes, endothelial cells and immune cells (Supplementary Figure S3C, Supporting Information).

Given that pH alone did not explain the cytotoxic effects of TEOA, we considered changes in medium tonicity due to high amounts of dissolved co‐initiator. We therefore analyzed cellular morphology in response to exposure to TEOA and observed a dramatic reduction in cell size that further progressed with time (Figure 3C). As this indicates osmotic stress due to elevated osmolyte concentrations, we measured media osmolality through freezing point depression (Figure 3D), which showed osmolality increasing with TEOA concentration. To test whether increased medium osmolality would indeed affect cell morphology, we analyzed cell morphology in response to TEOA exposure as a function of concentration and time as well as the recovery in normal growth medium after a maximum exposure of 60 min (Figure 3E). To isolate the effect of osmotic pressure from potential other cytotoxic effects, we used as a neutral osmolyte for comparison 400 Da polyethylene glycol (PEG). PEG is a non‐toxic, FDA‐approved polymer that is frequently used to increase osmolality in a controlled manner without affecting the pH (Supplementary Figure S3D, Supporting Information). We observed a time‐ and concentration‐dependent decrease both in cell area and aspect ratio (Figure 3F,G) for both PEG and TEOA, which slowly approached the respective control conditions during the 20 h recovery phase following treatment. The fact that the aspect ratio of recovered cells exceeded values observed before stimulation is due to a short adhesion window of 4 h before exposure to avoid niche formation by cell‐secreted ECM components that might create a protective layer. While cells fully regained their morphology after exposure to PEG, TEOA‐treated cells remained altered. Even at 2.5% TEOA, cells exhibited reduced cell area and aspect ratio 20 h post treatment, and at 5%, they remained rounded rather than adopting the spindle‐shaped fibroblast phenotype.

Next, we assessed viability in response to increasing PEG concentrations–again starting with hdFs and HEPG2 cells–and observed that only for very high concentrations of ≥ 20% (wt./vol. %), the viability was significantly reduced (Supplementary Figure S3E, Supporting Information). Since osmolyte concentration appeared not clearly indicative for the degree of stress imposed on cells, we analyzed HEPG2 viability in dependence of osmolality for PEG and TEOA and observed toxic effects for PEG above 1500 mOsm kg^−1^ and for TEOA above 601 mOsm kg^−1^ (Figure 3I). Since PEG did not affect the pH, we wondered whether the combination of tonicity and pH was responsible for the reduced viability of TEOA. When adjusting the PEG‐containing medium solution to pH 9.5, we observed a decrease in viability already for lower PEG concentrations which indicates that toxic effects of alkaline pH and tonicity converge. However, the viability decline was less pronounced than with TEOA, suggesting additional cytotoxic effects by TEOA itself. A likely explanation is the lysosomotropic function of small, membrane‐permeant weak bases such as ammonia.^[^ ^21^ ^]^ Thereby, the non‐protonated form diffuses across the cell membrane where the acidic environment of lysosomes leads to protonation and ion trapping. Lysosomal accumulation is particularly efficient for bases with a pK_a_ between 7 and 9 such as TEOA.^[^ ^22^ ^]^ Lysosomal leakage upon TEOA treatment was already demonstrated for isolated lysosomes and could explain the increased sensitivity of macrophages to TEOA exposure as their viability greatly depends on lysosomal integrity.^[^ ^23^, ^24^, ^25^ ^]^ We therefore simulated lysosomal alkalization through addition of ammonium chloride as a source of ammonia. We observed that a 60 min treatment with up to 250 mM ammonium chloride already decreased viability and metabolic activity of HEPG2 cells when directly supplemented into the medium (Figure 3I, Supplementary Figure S3F, Supporting Information). This decline was also observed with pH 9.5, hyper‐osmotic stress (15% PEG) and a combination of alkaline pH and osmotic pressure where NH_4_Cl supplementation reduced cell viability to levels comparable to 5% TEOA treatment (Figure 3I). This suggests that lysosomal alkalization, extracellular pH and hyperosmotic pressure act as independent but converging stressors and in combination explain the cytotoxic effects of TEOA. In contrast, pH adjustment that forced TEOA into its protonated, membrane‐impermeable form markedly reduced toxicity, since the compound was retained extracellularly (Supplementary Figure S3G, Supporting Information). As lysosomal alkalization and ion trapping imply a chronic impairment of cellular function even if cells survive the acute exposure, we validated the impact of a short TEOA pulse on long term functionality. Hereby, cell growth over 7 days was significantly reduced already for concentrations of 2.5% for HEPG2 and hdF cells while osteogenic differentiation of hMSCs was completely suppressed after 14 days (Figure 3J, Supplementary Figure S3H–J, Supporting Information). This underlines the long‐term impairment of cell function even for conditions that display high initial cell viability.

Overall, our findings demonstrate that TEOA's poor biocompatibility arises from the combined effects of hyper‐osmotic stress, lysosomotropic function and extracellular alkalinity. While high osmolality alone can impact cell morphology, lysosome alkalization exacerbates cytotoxicity, particularly in lysosome‐dependent immune cells such as monocytes. These converging stress factors fundamentally limit TEOA's suitability as a co‐initiator.

### 2.3 BisTris Serves as an Alternative Co‐Initiator for Printing at Physiological pH

**Figure 4:**
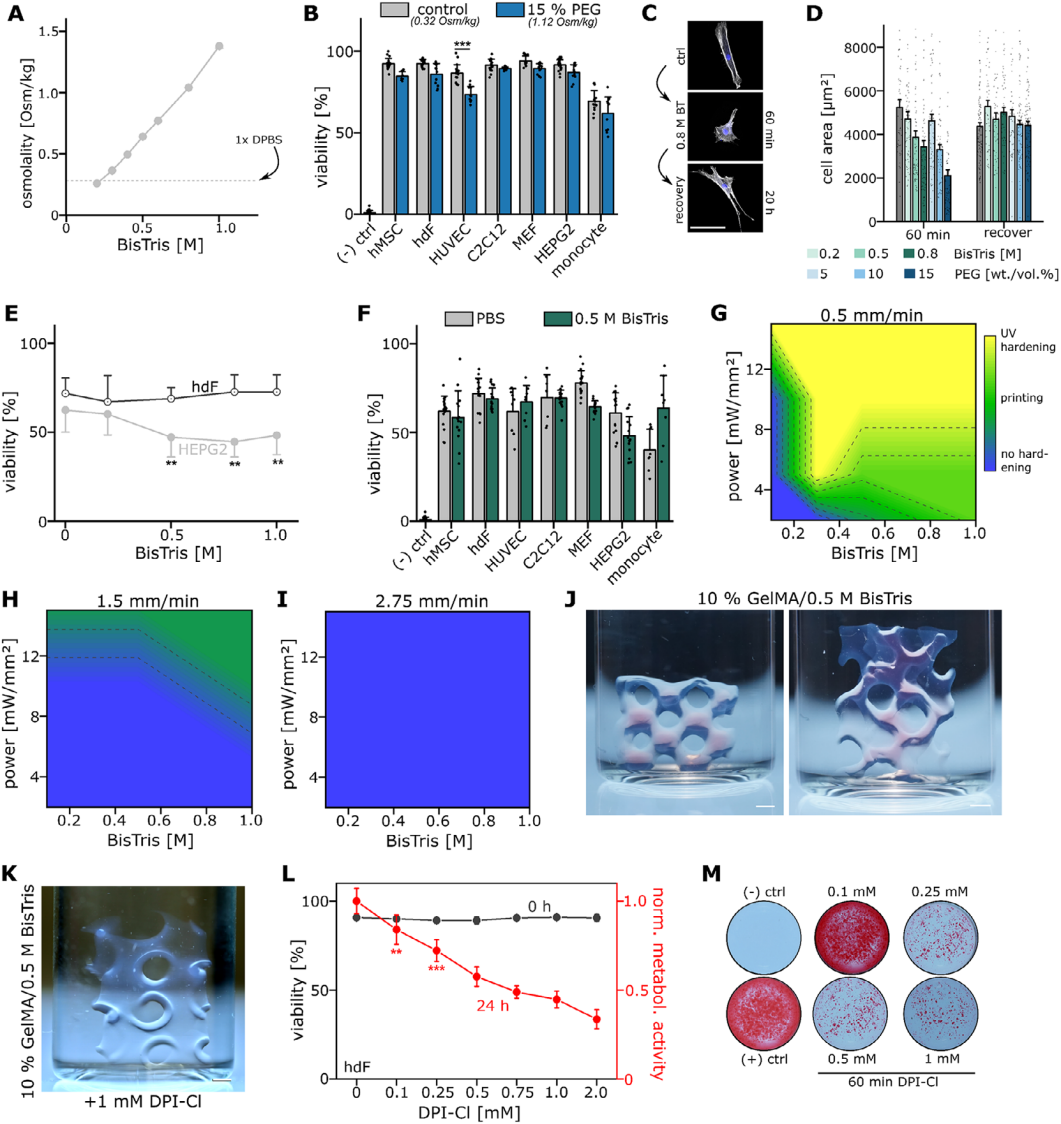
BisTris serves as a functional co‐initiator at physiological pH. A) Medium osmolality measured by freezing point depression of a titrated BisTris solution (concentration between 0.1 and 1 M) at pH 7.4. The gray dashed line indicates osmolality of PBS. B) Viability (% of live cells) of hMSCs, hdFs, HUVECs, C2C12, MEFs, HEPG2 and primary human monocytes after 60 min of exposure to 15% PEG diluted in expansion medium. A 0.1% Saponin sample served as negative control for reference. N ≥ 8 from at least two independent experiments. C) Confocal images of hdF cells exposed to 0.8 M BisTris for 60 min and subsequent recovery by changing the medium to fresh expansion medium. Cytoskeleton stained in white (actin), nuclei in blue. Scale bar 100 µm. D) Quantification of cell area of hdFs after exposure to a varying concentration either of BisTris (0.2–0.8 M) or PEG (molecular weight: 400 g mol^−1^, 5–15 wt./vol.%) for 60 min to indicate cellular shrinkage due to hyper‐osmotic pressure. Recovery of cell area to control levels was quantified 20 h after exchanging the medium to fresh expansion medium. Data are depicted as Mean + SEM. N > 50 cells of at least two independent experiments E) Viability (% of live cells) of HEPG2 (light gray) and hdF (dark gray) cells shown as a function of BisTris concentration ranging from 0.2 to 1 M after 60 min of exposure. N ≥ 8 from at least 2 independent experiments. Statistics tested against 0% BisTris control. F) Viability (% of live cells) of hMSCs, hdFs, HUVECs, C2C12, MEFs, HEPG2 and primary human monocytes after 60 min of exposure to 0.5 M BisTris. A 0.1% Saponin sample served as negative control and PBS as positive control for reference. N ≥ 8 from at least two independent experiments. G–I) Heatmaps depicting printability as a function of power input (y axis) and BisTris concentration (x axis) for slow (0.5 mm min^−1^), medium (1.5 mm min^−1^) and fast (2.75 mm min^−1^) print speeds. Color code indicates UV hardening (yellow), successful printing (green) and no object hardening (blue) with interpolation in between these 3 states. J) Example print objects (gyroids) printed via Xolography using 10% (wt./vol.) GelMA and 0.5 M BisTris as co‐initiator at physiological pH. Scale bar 2 mm. K) Example print using 10% GelMA and 0.5 mM BisTris as co‐initiator as base formulation which was additionally supplemented with 1 mM DPI‐Cl. Scale bar 1 mm. These prints were generated using DPI‐Cl to demonstrate resolution potential, but this formulation was found to be cytotoxic and is not recommended for use in viable bioinks. L) Quantification of cell viability of hdFs which were exposed to a varying concentration of DPI‐Cl for 60 min diluted in growth medium. The viability was directly measured after treatment (gray line, left axis). Overlaid red line depicts normalized metabolic activity (% of control) measured via Alamar Blue 24 h after DPI‐Cl treatment. N = 8 of two independent experiments. M) Representative Alizarin Red stains after 14 days of osteogenic differentiation of hMSCs which were treated for 60 min with a varying concentration of DPI‐Cl prior to osteogenic stimulus. Statistics via a two‐sided Mann‐Whitney‐U test. A value of <0.05 was considered as statistically significant. Individual significance levels are indicated as follows: *: *p* < 0.05, **: *p* < 0.01, ***: *p* < 0.001.

Given the limitations of TEOA, we systematically evaluated BisTris as an alternative, biocompatible co‐initiator candidate. The lower basicity (pK_a_ = 6.48) allows printing at physiological pH as 91.6% of all BisTris molecules are present in the desired non‐protonated form at pH 7.4 (Supplementary Figure S4A, Supporting Information). Furthermore, BisTris did not display lysosomotropic effects which can be attributed both to its lower pK_a_ and its higher molecular weight and polarity that prevent passive diffusion into intracellular compartments.^[^ ^22^, ^23^ ^]^ Thus, unlike TEOA, BisTris combines the required chemical reactivity with intrinsic biocompatibility. However, a recent study reported reduced metabolic activity for BisTris‐containing formulations which might be caused by an excessive hyper‐osmotic shock that likely exceeded physiologically tolerable levels.^[^ ^15^ ^]^ At pH 7.4, BisTris exhibited a concentration‐dependent tonicity that increased to 1382 mOsm kg^−1^ for a 1 M solution (**Figure** **4**B). When considering the isolated effect of osmolality, for a time window of 60 min, all analyzed cell types showed only mild reduction in viability for an osmolality of 1100 mOsm kg^−1^ at pH 7.4 (associated with a concentration of 15% PEG) or 0.8–1 M BisTris (Figure 3J). This range is close to values found in vivo where osmolality can reach ≈450 mOsm kg^−1^ in intervertebral discs and ≈1200 mOsm kg^−1^ in gastrointestinal villi in contrast to the tightly regulated blood serum levels of ≈290 mOsm kg^−1^.^[^ ^26^ ^]^ We validated the effect on cell morphology as an early indicator of osmotic stress and observed the expected reduction in cell area for 0.8 M BisTris, which was completely reversed upon exchange to the normal growth medium (Figure 4C,D). Hence, BisTris, like TEOA, creates a hyper‐osmotic environment but can be used at a cell‐friendly, physiological pH and without lysosomotropic effects.

We then assessed the biocompatibility of BisTris. Initially, we quantified cell viability of the more sensitive HEPG2 and more resilient hdF cells as a function of BisTris concentration in a pure solution without additional optically active supplements which mimics the necessary conditions for Xolography and volumetric bioprinting in general. Since already a 0.2 M BisTris solution has an osmolality comparable to PBS (Figure 4B), we did not supplement additional ions as those would increase medium tonicity and thereby constrain the usable range before reaching cytotoxic tonicity levels. Due to the lack of nutrients in the BisTris solution, the viability of cells overall decreased to a level comparable to PBS but showed only a mild decline in HEPG2 cells at concentrations up to 1 M (Figure 4E) which reacted more sensitive to osmolality changes under the before‐discussed conditions. Notably, cell viability exceeded that of a 2.5% TEOA solution (Figure 2F). This trend was further confirmed for 0.5 M BisTris across all additional cell types, which exhibited viability values similar to those observed in PBS (Figure 4F). This identifies 0.5 M BisTris as a biocompatible concentration.

In a next step, we screened printing conditions using a 10% GelMA solution and a varying BisTris concentration to check whether printing was possible at concentrations that do not compromise cell viability (<1 M BisTris). We tested three different print speeds ranging from slow (0.5 mm min^−1^) to medium (1.5 mm min^−1^) and high (2.75 mm min^−1^) for a light sheet power output ranging from 2 to 15 mW mm^−^^2^ and a BisTris concentration ranging from 0.1 M to 1 M (Figure 4G–I). For the low printing speed 0.1 M BisTris was insufficient to allow object formation. In contrast, starting with 0.3 M, we observed object formation and even partial UV hardening for high light sheet intensities as a result of UV light absorption by the active merocyanine DCPI species. At higher print speeds, a higher BisTris concentration was necessary and for the highest print speed of 2.75 mm min^−1^ we did not observe any conditions that resulted in the formation of polymerized objects. The reported DCPI kinetics (Figure 1B,C) imply that successful printing depends on a sufficient temporary concentration of merocyanine active species that needs to be formed. This is not the case for high print speeds where each voxel is exposed for ≈1 s, while for 0.5 mm min^−1^, the exposure is 6 s (assuming light sheet thickness of 50 µm). Together these findings establish that a BisTris concentration of 0.3–0.5 M is sufficient at low print speeds, balancing both polymerization efficiency and biocompatibility. We used this printing condition to realize various objects in order to demonstrate the printing capacities of the identified co‐initiator system for a 10% GelMA solution (Figure 4J). We show that complex geometries and smooth surfaces can be realized with high precision with Xolography using a biocompatible hydrogel matrix (here: gelatin) in combination with the identified biocompatible co‐initiator concentration. We further assessed the capable printing resolution and observed retrievable features in fully processed objects of down to 100 µm using defined feature resolution plates and geometries which is comparable to other volumetric bioprinting techniques but lower than previous Xolography benchmarks (≈ 20 µm) that used TEOA/Diphenyliodoniumchloride (DPI‐Cl)‐containing formulations (Supplementary Figure S4B, Supporting Information).^[^ ^11^, ^27^ ^]^ Such printed objects revealed a stiffness of 190 ± 12 Pa (storage modulus G’) which is within the range typically reported for soft, cell‐compatible GelMA hydrogels (0.1–30 kPa) depending on polymer concentration, methacrylation degree, and curing conditions.^[^ ^28^, ^29^ ^]^

However, as the achievable printing speed and resolution were lower compared to preceding studies, we explored the use of DPI‐Cl as a reactivity booster which was recently reported to improve print performance in a TEOA‐containing system.^[^ ^27^ ^]^ Indeed, we also observed improved reactivity (7 mJ mm^−^^2^ versus 20 mJ mm^−^^2^ without DPI‐Cl) and print fidelity with supplementation of 1 mM DPI‐Cl in a BisTris printing system (Figure 4K). Particularly smaller features such as the rim of opening pores exhibited a higher fidelity. Since diarylhalonium salts are known to irreversibly inhibit oxidoreductases (e.g., succinate dehydrogenase), we not only assessed viability, but also metabolic activity and differentiation potential as a function of DPI‐Cl concentration.^[^ ^30^ ^]^ While the viability was unaffected and independent of DPI‐Cl concentration after 60 min of exposure, we observed a reduction in the metabolic activity already for concentrations as low as 0.1 mM (Figure 4L).^[^ ^27^ ^]^ Furthermore, we detected a loss of differentiation potential after 2 weeks of osteogenic differentiation of hMSCs for concentrations of DPI‐Cl exceeding 0.1 mM (Figure 4M) while the recent study introduced a minimal concentration of 0.5 mM to boost reactivity and a maximum working concentration of 3.1 mM.^[^ ^27^ ^]^ These findings suggest that, despite improving printability, DPI‐Cl introduces severe and lasting impairments to cellular metabolic function, which are not captured by short‐term viability assessments alone – confirming the described oxidoreductase inhibition mechanism.^[^ ^30^ ^]^ Therefore, while DPI‐Cl can serve as a critical component in cell‐free material printing, its application in living systems needs to be carefully considered, particularly when combined with TEOA. Future investigations should therefore explore alternative biocompatible agents.

### 2.4 Nutrient Supplementation Increases Biocompatibility of BisTris‐Based Bioinks

**Figure 5:**
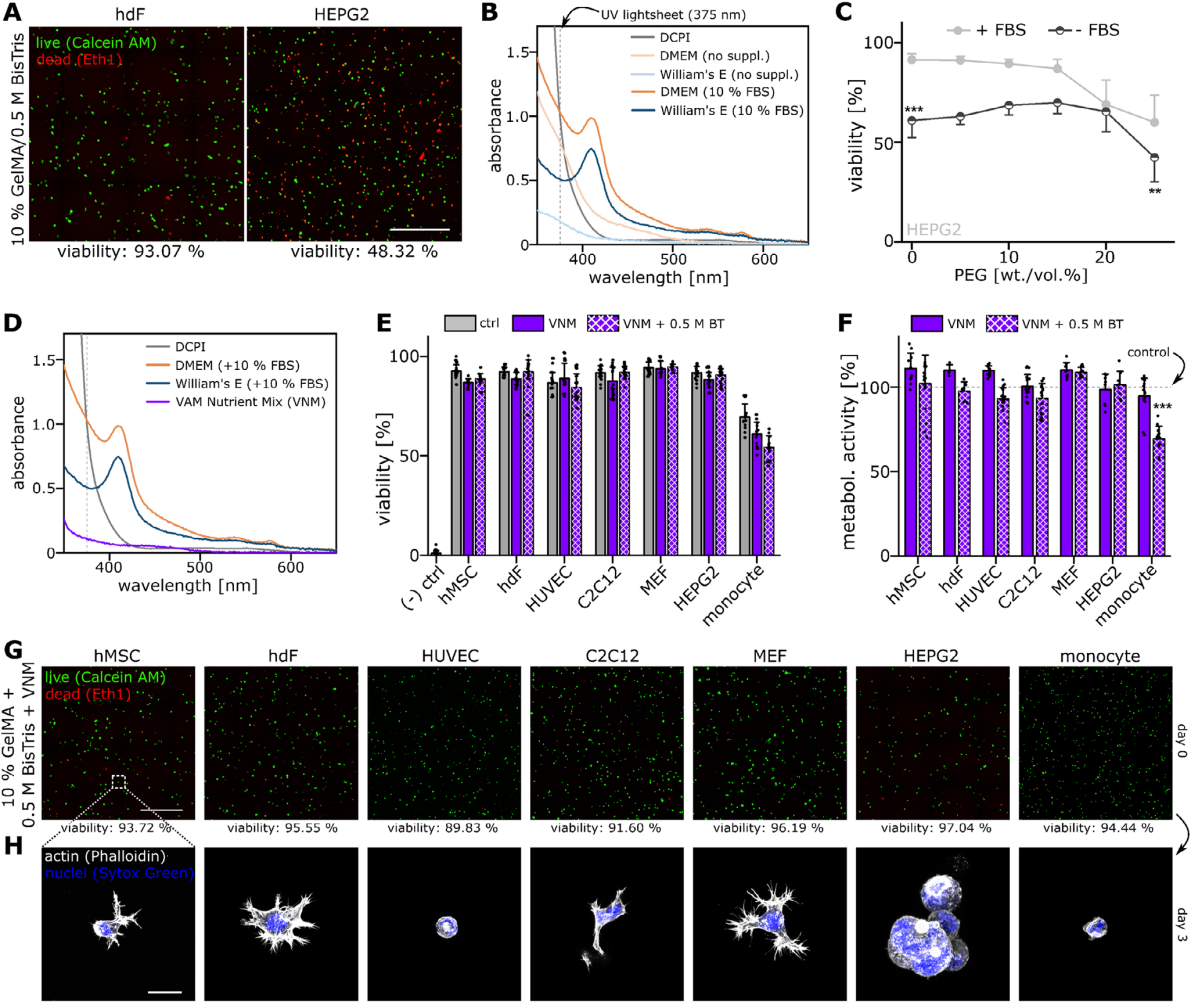
A low‐absorbing nutrient mix enhances cell viability during printing. A) Confocal Images of hdF (left) or HEPG2 (right) cells printed via Xolography using 10% GelMA and 0.5 M BisTris as resin formulation. Viability was visualized by Calcein‐AM (live, green)/Eth1 (dead, read) staining. Scale bar 500 µm. B) Absorbance spectra of DMEM and William's E basal media as well as the respective growth medium formulation containing 10% FBS. Absorbance of the DCPI (gray) and dashed line for indication of light sheet activation wavelength serve as reference. C) Viability (% of live cells) of HEPG2 cells shown as a function of PEG concentration ranging from 5–25% (wt./vol.) after 60 min of exposure. PEG was either diluted in full growth medium (containing 10% (vol./vol.) FBS, light gray) or basal medium (without FBS, dark gray). N ≥ 8 from at least two independent experiments. D) Absorbance spectra of VAM Nutrient Mix (VNM, 1× formulation, xolo GmbH) compared to absorbance of William's E and DMEM growth media. Absorbance of the DCPI (gray) and dashed line for indication of light sheet activation wavelength serve as reference. E) Viability (% of live cells) of hMSCs, hdFs, HUVECs, C2C12, MEFs, HEPG2 and primary human monocytes after 60 min of exposure to VNM (1× ‐ diluted in 1× PBS) or 1× VNM diluted in 0.5 M BisTris. A 0.1% (wt./vol.) Saponin sample served as negative control and PBS as positive control for reference. N ≥ 8 from at least two independent experiments. F) Metabolic activity 24 h post treatment (normalized to untreated control cells) of cells which were exposed to VNM (1× ‐ diluted in 1× PBS) or 1× VNM diluted in 0.5 M BisTris. N ≥ 8 from at least two independent experiments. G) Confocal images (maximum projection) depicting cell viability after printing via Xolography using 10% GelMA, 0.5 M BisTris and VNM supplement. Viability was visualized by Calcein‐AM (live, green)/Eth1 (dead, read) staining. Scale bar 500 µm. H) Zoom‐in confocal images of fixed samples which were stained for cytoskeleton (actin, white) and nuclei (blue) after 3 days of culture. Scale bar 20 µm. Statistics via a two‐sided Mann‐Whitney‐U test. A value of <0.05 was considered as statistically significant. Individual significance levels are indicated as follows: *: *p* < 0.05, **: *p* < 0.01, ***: *p* < 0.001.

Following the establishment of a non‐toxic co‐initiator system, further optimization of cellular viability and functional maintenance during printing was necessary–particularly when actual printing conditions introduce additional stress factors such as UV exposure. Especially sensitive cells such as endothelial cells, monocytes or hepatocytes react to even small and short deprivations of nutrients. Although the printing itself takes only a few seconds to minutes, the overall process is still time‐consuming due to post‐processing considering liquid handling, solidification, printing, dissolving, washing and curing–particularly when processing more than one sample at once. As a result, a full printing process resulted in a reduced viability of 48% for HEPG2 cells, while fibroblasts remained mostly viable (**Figure** **5**A). This is in line with previous findings for CAL printing, where iPSC‐derived hepatocytes in suspension showed low viability in a pure GelMA/PBS environment while multi‐cellular organoids displayed a higher viability.^[^ ^11^ ^]^ It further implies that more sensitive cells require nutrient supply during printing to maintain survival and functionality. Standard media, especially when supplemented with FBS, show a high UV absorbance (Figure 5B). Following Beer‐Lambert law, this leads to an inhomogeneous UV light sheet and visible light profiles across the buildroom that will further result in inhomogeneous degrees of conversion (Supplementary Figure S5A, Supporting Information). We further observed that the supplementation of FBS is necessary to maintain a high viability of HEPG2 cells both in an iso‐ and a hyper‐osmotic environment (Figure 5C).

To provide conditions as found in full cell growth media without the unwanted optical interference, we used an advanced nutrient mix supplement (VAM Nutrient Mix (VNM), xolo GmbH) that was specifically designed for volumetric printing applications. We analyzed absorbance and observed that the formulation exhibited a highly reduced absorption of more than 90% transmittance (compared to DMEM supplemented with 10% FBS, 1 cm path length) both in the UV and visible light range (Figure 5D). Next, we measured the viability and metabolic activity of all analyzed cell types both, in the presence of VNM and of a mixture of VNM and 0.5 M BisTris (Figure 5E,F). Notably, we observed unchanged viability measures for all analyzed cell types both in the presence of VNM and 0.5 M BisTris together with VNM only compared to controls. The only noteworthy exception was monocytes that showed reduced metabolic activity when exposed to a combination of BisTris and VNM. As this is not the case for cells in the VNM only condition, it can be assumed that the formulation is not optimal to support viability in hyper‐osmotic environments–caused by BisTris in this particular case. Generally, supplementing an advanced nutrient formulation restores cell viability and metabolism in a hyper‐osmotic environment to control levels while reducing optical interference (Supplementary Figure S5B, Supporting Information). Furthermore, while even short incubation of hMSCs with 2.5% TEOA interfered with osteogenic differentiation over 2 weeks, we did not observe any influence when applying the optimized print mix in the same setup neither on proliferation nor osteogenic differentiation (Supplementary Figure S5C–F, Supporting Information).

To finally prove that the optimized formulation results in high viability during printing, we observed cell viability after Xolography printing using live‐dead staining for all presented cell types (Figure 5G). Most importantly, unlike for a nutrient‐free printing setup that showed varying viability (Figure 5A), we observed a strongly improved viability for all analyzed cell types ranging from 89.8% (HUVECs) to 94.7% (HEPG2). Monocyte viability improved in 3D conditions, likely due to gelatin adhesion sites supporting integrin‐mediated cell survival, enhancing viability in suspension cultures. Cytoskeletal staining of encapsulated cells after 3 days further indicated the onset of spreading, elongation or clustering of cells related to the musculo‐skeletal system while monocytes, HEPG2 and HUVEC cells remained round in morphology (Figure 5H).

In sum, the optimized formulation ensures high viability in a BisTris‐based print mix by balancing osmotic conditions, eliminating optical interference, and providing essential nutrients.

### 2.5 Long Term Survival and Tissue Formation in Xolography‐Printed Structures

**Figure 6:**
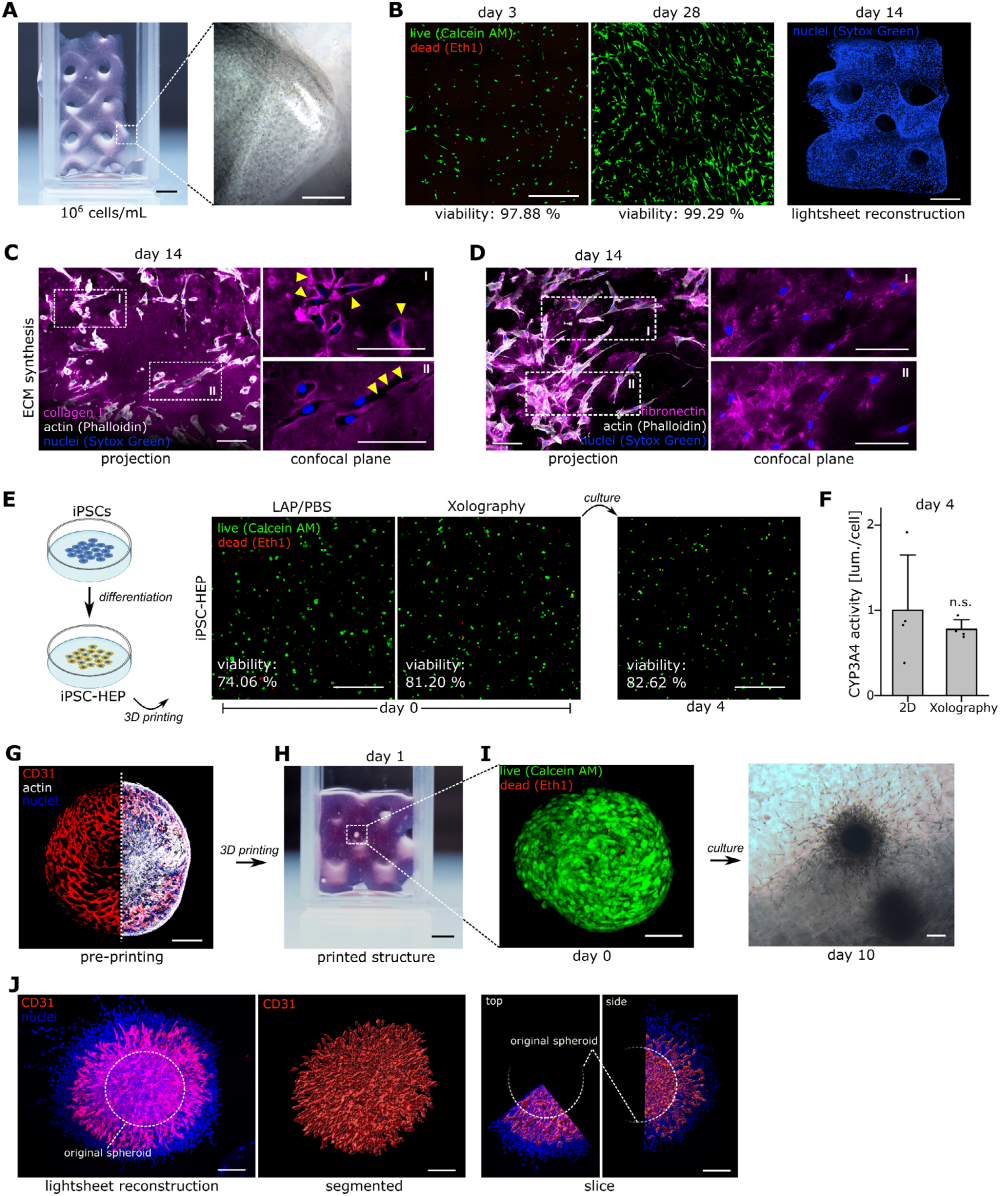
An optimized printing setup allows functional bioengineering. A) Example print object (gyroid) printed via Xolography using 10% GelMA and 0.5 M BisTris as co‐initiator at physiological pH with 10^6^ cells mL^−1^ of primary hdFs. Zoom‐in depicts phase‐contrast image with 10× magnification. Scale bar 2 mm. B) left: live/dead stains of primary hdFs 3 and 28 days after printing. Live cells are depicted in green (Calcein AM), while dead cells are depicted in red (Eth‐1). Scale bar 500 µm. Right: light sheet reconstruction of hdFs cultured for 14 days inside Xolography‐printed gyroid constructs. Blue: cell nuclei. Scale bar 2 mm. C) Confocal images of primary hdFs after 14 days of culture in GelMA‐printed constructs. Left depicts a maximum projection while zoom‐in display single confocal planes. Magenta shows human collagen type I, white F‐actin and blue the cell nuclei. Yellow arrows depict cavities created through matrix degradation. Scale bar 100 µm. D) Confocal images of primary hdFs after 14 days of culture in GelMA‐printed constructs. Left depicts a maximum projection while zoom‐in display single confocal planes. Magenta shows human fibronectin, white F‐actin and blue the cell nuclei. Scale bar 100 µm. E) Live/dead stains of iPSC‐derived hepatocytes either embedded into an LAP‐crosslinked hydrogel or Xolography‐printed construct directly after printing and 4 days after printing in the case of Xolography‐printed constructs. Scale bar 500 µm. F) Quantification of CY3A4 activity 4 days after printing in comparison to 2D TCP‐seeded controls. The signal was normalized to the DNA content indicative for the amount of cells. G) Confocal image of a spheroid cross‐section after 2 days of culture in non‐adhesive well plates. CD31 (red) marks endothelial cells, F‐actin (white) the cytoskeleton and nuclei are depicted in blue. Scale bar 200 µm. H) Image of a printed gyroid structure depicting the incorporation of spheroids into printed objects. Scale bar 2 mm. I) Left: confocal image of a live/dead stained spheroid right after printing via Xolography. Green shows live, red dead cells. Right: Phase contrast image after 10 days of culture displaying outgrowth of cells into the surrounding hydrogel. Scale bar 100 µm. J) 3D reconstructions of light‐sheet imaged samples displaying cell nuclei (blue) and CD31‐positive cells (red). White dashed line indicates the original spheroid dimensions. Scale bar 200 µm. Statistics via a two‐sided Mann‐Whitney‐U test. A value of <0.05 was considered as statistically significant. Individual significance levels are indicated as follows: *: *p* < 0.05, **: *p* < 0.01, ***: *p* < 0.001.

To demonstrate the potential for bio‐medical applications, we continued to examine cellular function and long‐term viability in printed complex environments (**Figure** **6**A). At cell densities of up to 1 × 10⁶ cells mL^−1^, printed geometries remained indistinguishable from acellular references, consistent with previous CAL‐based volumetric printing results by Bernal et al., who demonstrated accurate reproduction of 500 µm features at identical cell densities without the need for refractive‐index matching.^[^ ^11^ ^]^ Within this regime, light scattering remains limited and does not measurably affect shape fidelity. We started by observing cell viability of fibroblasts over a time course of up to 4 weeks (Figure 6B). We found that, starting from an already high cell viability of 94%, cells showed signs of spreading and elongation at day 3. After 4 weeks of culture, the cell density has increased and cells showed a spread and elongated morphology typical for successful adhesion to the matrix of the printed construct. The viability was 99% and we observed an increased cell density, suggesting a continuous proliferation of cells inside the biomaterial and very limited apoptosis. In addition, we followed up on extracellular matrix (ECM) production. Particularly fibroblasts are known for their tissue‐forming potential as they secrete fibronectin and different relevant types of collagens.^[^ ^31^ ^]^ In vitro, this process starts as early as few days after seeding and peaks ≈14 days.^[^ ^32^ ^]^ We therefore followed up on fibronectin and collagen deposition after 2 weeks of culture and could observe that cells were embedded in a diffuse but dense layer of type I collagen while creating staining‐negative cavities. Altogether this created snail‐like trails within the material (Figure 6C). The reason for the restriction of cell mobility to individual trails is the dense photochemical crosslinking of the gelatin bio‐polymer which needs to be enzymatically degraded in order to allow cell spreading and migration. The different speed at which this happens is also reflected in morphological differences between individual cells which were partially found rounded and less spread while others were fully elongated. This generally limits the deposition of new ECM to a confined space that is expected to gradually increase with progressing material degradation. Furthermore, we observed cell‐derived fibronectin– also locally aligned around the cell bodies (Figure 6D). In contrast to collagen I, fibronectin displayed a clearer fibrillar character which might be an indication of local tensioning. Together, while we could observe viability, spreading and ECM production, the photochemical crosslinking of the gelatin bio‐polymer confined this process to a region around individual cells without a collective, tissue‐like character yet.

Beyond building tissue models that rely on the production of ECM, other models rely on the proper function and maintenance of a distinct differentiation/maturation profile after printing. A prominent example is the fabrication of liver models that rely on the functional integrity of hepatic cells. These models are expected to serve as powerful tools for patient‐specific disease models or drug screening approaches.^[^ ^33^ ^]^ As a proof‐of‐principle we used iPSC‐derived hepatocytes (iPSC‐HEP) and printed them into a 3D environment via Xolography. As there are indications that serum osmolality correlates with the severity of Hepatic encephalopathy, we wondered whether the hyper‐osmotic printing conditions are harmful for these cells that can be considered more vulnerable as the already introduced HEPG2 cell line.^[^ ^34^ ^]^ We compared cell viability in Xolography‐printed samples to Lithium‐Phenyl‐2,4,6‐trimethylbenzoylphosphinat (LAP)‐cured hydrogels (which is used in many photo‐curing approaches) and found viability to be slightly higher in Xolography‐printed samples (Figure 6E). Noteworthy, while the Xolography printing was based on the optimized bioink formulation, LAP‐based photo‐curing was conducted in PBS as it is usually observed in most protocols.^[^ ^11^, ^33^ ^]^ Hence, our data further suggest that supplementation of nutrients can be beneficial to preserve cell viability during Xolography printing and its hyper‐osmotic environment compared to usually nutrient‐free but iso‐osmotic ink formulations. We further followed up on cell viability over a time window of 4 days with no considerable decline in viability. We further measured Cytochrome P‐450 3A4 (CYP3A4) activity which is the most abundant and also relevant enzyme for metabolizing drugs and serves as a critical marker for assessing maintenance of hepatocyte function.^[^ ^35^, ^36^ ^]^ As we did not expect differences between Xolography‐printed and LAP‐cured hydrogels, we compared the enzymatic activity to TCP‐plated cells (2D) for which we only observed a mild, but non‐significant reduction, further confirming the biocompatible printing setup (Figure 6F).

While so far, the applicability toward individual cells was shown, we finally wanted to showcase the potential of Xolography for fabrication of advanced model systems. A major challenge in tissue engineering is the generation of vascularized constructs. As this usually requires high cell densities, the usage of pre‐vascularized spheroids or organoids is a promising way to overcome the need of working with high cell densities in light‐based printing as they act as scattering particles.^[^ ^37^, ^38^, ^39^ ^]^ We therefore combined hdF and HUVECs that can undergo spontaneous self‐assembly in non‐adherent environments into compact spheroids with a dense vascular plexus (Figure 6G). These spheroids were then printed into GelMA constructs and vascular outgrowth was observed with light sheet imaging (Figure 6H–J). Here, we could observe the outward migration both of fibroblasts and vascular structures following a partial cellular outgrowth of the formed spheroids. Importantly, this shows that multicellular structures can not only be successfully integrated into Xolography‐printed hydrogel constructs but in addition stromal and endothelial cells are engaged in the formation of an outgrowing vascular network.

In sum, these above examples verify the potential of Xolography for the fabrication of advanced and functional tissue models for drug screening or personalized disease modeling.

## 3 Conclusion 

Xolography is a novel and promising volumetric additive manufacturing technique.^[^ ^14^, ^20^, ^40^, ^41^ ^]^ Previous studies have demonstrated the technical feasibility of Xolography for bioprinting, achieving sub‐20 µm resolution and localized stiffness modulation in hydrogel systems.^[^ ^15^, ^27^ ^]^ However, these advances were primarily driven by photochemical and optical optimization, whereas appropriate conditions for high viability bioprinting were largely undefined. As a result, bioink formulations that were reported as compatible in short‐term viability assays ‐ particularly those based on triethanolamine (TEOA) and diphenyliodonium chloride ‐ are now shown to compromise long‐term metabolic activity and functional performance.^[^ ^27^ ^]^

**Figure 7:**
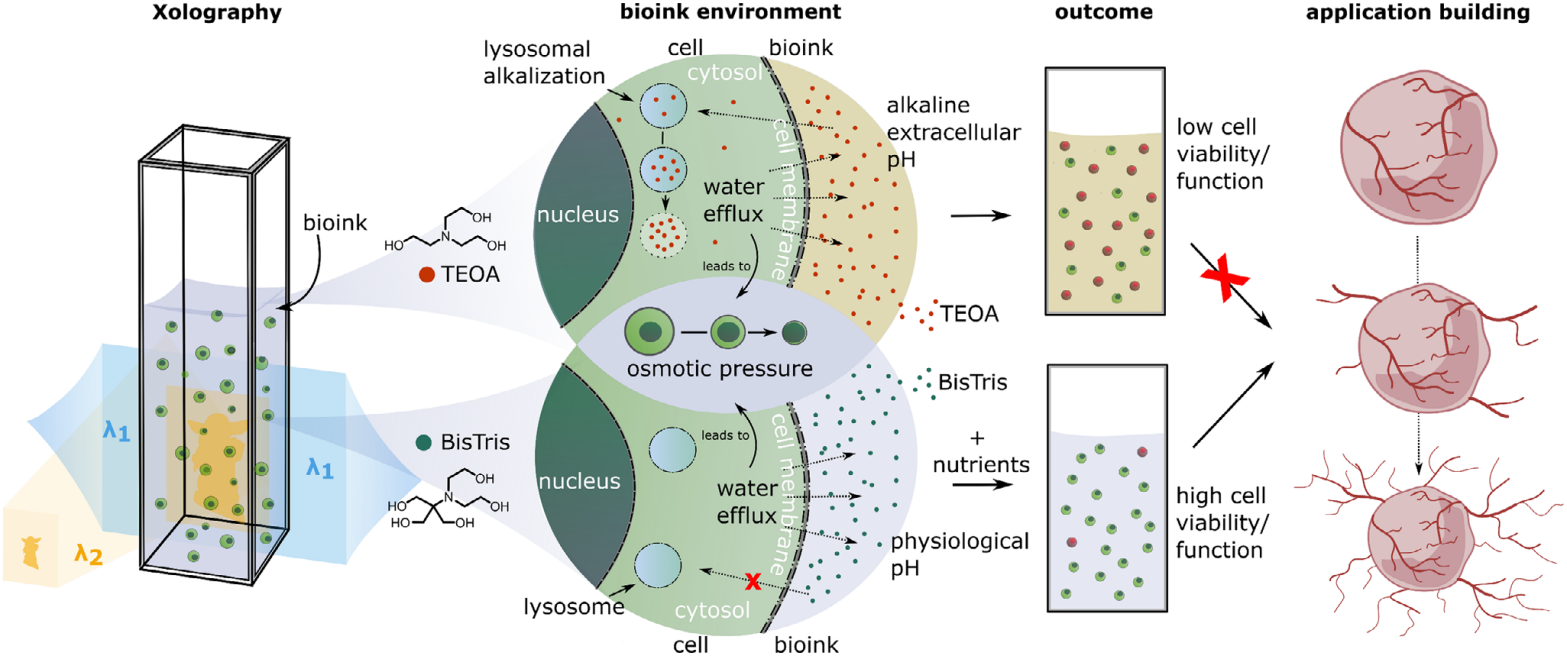
Schematic illustration of the biochemical constraints in Xolography bioinks. During volumetric printing, cells are embedded in bioinks that differ fundamentally in their co‐initiator chemistry. Triethanolamine (TEOA) formulations expose cells to three converging stressors – extracellular alkalinity, osmotic water efflux, and lysosomal alkalization – which in combination severely impair viability and long‐term function. In contrast, BisTris‐based formulations avoid lysosomal trapping and extracellular alkalinity, leaving osmotic pressure as the main constraint. Supplementation with nutrients further supports cell function at physiological pH. As a result, TEOA‐based systems lead to low viability and functional failure, whereas BisTris‐based systems sustain high viability and enable functional tissue building, exemplified by sprouting vascular spheroids.

Unlike most light‐based bioprinting techniques, Xolography requires tertiary amines as co‐initiators which act as weak bases. The present study therefore provides the first systematic definition of the biochemical design space of Xolography‐based bioprinting, identifying alkaline pH, lysosomal alkalization, and hyperosmotic pressure as independent but converging stressors governing cytocompatibility. All three stressors coincide in TEOA‐based systems which inherently limit TEOA's suitability for bioprinting applications. In particular, lysosomotropic ion trapping of TEOA emerged as a persistent stressor that impaired cell function beyond the short exposure window.^[^ ^21^ ^]^ The low pK_a_, high polarity and molecular weight of BisTris, however, eliminate two of these stressors, leaving hyperosmotic pressure as the only remaining constraint that limits the maximum concentration tolerated by cells during printing.^[^ ^22^ ^]^ Such residual cytotoxic effects can further be mitigated by a tailored nutrient formulation that does not compromise the optical transparency required for efficient polymerization. Our design framework is therefore capable to reconcile previously conflicting observations on cell viability and functionality in volumetric printing and establishes the biological “safe operating space” within which Xolography can be applied reliably. In doing so, our work transforms Xolography from a primarily photochemical concept into a biochemically grounded biofabrication platform, enabling reproducible and biologically credible printing of cell‐laden constructs (**Figure** **7**). With optimized formulations, we achieved optical feature resolutions of down to 100 µm which are comparable to other volumetric printing techniques but lower than preceding work on Xolography reporting minimum features of 20 µm that relied on toxic enhancers.^[^ ^11^, ^15^, ^27^ ^]^ However, this cell biology‐centered approach followed here provides a stable foundation from which reactivity and resolution can be systematically improved, for example through contrast enhancers and temperature control.^[^ ^42^ ^]^

A key constraint to overcome in volumetric printing is scattering of light by cells. At the cell densities used in this study (≤1 × 10⁶ cells mL^−1^), such effects remained negligible and did not measurably affect print fidelity, consistent with previous CAL‐based volumetric printing work, who reported accurate reproduction of 500 µm features at similar cell concentrations.^[^ ^11^ ^]^ At densities above 2.5 × 10⁶ cells mL^−1^, the study reported loss of feature resolution due to a significant increase in light scattering. Yet, overcoming this density barrier without compromising resolution is essential for advanced tissue models. Two complementary strategies are emerging: refractive index (RI) matching of cells with the surrounding bioink, and the use of multicellular structures (e.g., spheroids or organoids) to locally increase density without excessive scattering. The potential of these approaches has been demonstrated for other volumetric printing techniques and underscores the relevance of our biochemical framework as the prerequisite for implementing such optical strategies in Xolography.^[^ ^11^ ^]^

Together, these advances position Xolography not only as a technically feasible but as a biologically credible platform. Refining bioinks for high viability, print performance, and function will enable the fabrication of more complex tissue and organ models, and ultimately bridge the gap between experimental research and biomedical applications.

## 4 Experimental Section

### Absorption Measurements

Absorption spectra of the here used DCPI (DCPI 5002, Xolo GmbH) and the corresponding half‐life values were characterized as described previously.^[^ ^14^ ^]^

Absorption spectra of media components were measured using a Varian Cary 50 Scan UV–vis Photometer in 1 cm UV cuvettes (Brand GmbH, #759 170) which were also used for Xolography 3D printing. Raw absorption values were normalized to the absorption value of the DCPI at 375 nm.

### Osmolality Measurements

Osmolality of a solution was determined by freezing point osmometry (OSMOMAT Auto, Gonotec). Prior to measurement, solutions were equilibrated at room temperature and briefly vortexed. Each measurement used a sample volume of 50 µL. For each solution, three samples were measured.

### Cell Culture

All cells that were harvested from primary human material (tissue, blood) were isolated in accordance to a grant (EA2/152/24) submitted to the Ethics Committee of the Charité – University Hospital Berlin. All patients gave their written consent prior to obtaining material.

Primary human dermal fibroblasts (hdFs) were isolated from human skin biopsies through outgrowth culture. Cells were used between passages 4 and 9 and were cultured in Dulbecco's modified Eagle's medium, high glucose (DMEM, Thermo Fisher, # 11 965 092) supplemented with 10 vol.‐ % fetal bovine serum (FBS Superior, Sigma Aldrich, #S0615), 1 vol.‐ % Penicilin/Streptomycin (P/S, Bio & Sell, BS.A2212), 1 vol.‐ % non‐essential amino acids (NEAA, Bio & Sell, BS.K0293) at 37 °C with 5% CO_2_ in a humidified incubator. HdFs were passaged upon reaching confluency using 1× Trypsin/EDTA (Sigma Aldrich, T4174).

Primary human Mesenchymal Stromal Cells (hMSCs) were isolated from bone marrow and used in Passages 3–4. Cells were cultured in DMEM (low glucose, Thermo Fisher, #11 054 020) supplemented with 10% FBS, 1% P/S and 1 vol.‐ % GlutaMAX (Thermo Fisher, #35 050 038). HMSC were passaged at 80–90% confluency using 1× Trypsin/EDTA.

C2C12 and HEPG2 cells were cultivated in the same growth medium as hMSCs and passaged before reaching confluency using 1× Trypsin/EDTA. C2C12 cells were a generous gift from the lab of Petra Knaus.

Mouse Embryonic Fibroblasts (MEFs) were cultured in DMEM (high glucose, Thermo Fisher, #11 965 092), supplemented with 15% FBS, 1% P/S and 2 mM sodium pyruvate. Cells were passaged using 1× Trypsin/EDTA upon reaching confluency. Cells were a generous gift from the lab of Georg Duda.

Human Umbilical Vein Endothelial Cells (HUVECs) were purchased from Lonza (pooled donor, C2519A) and cultivated in Endothelial Cell Growth Medium (EGM) consisting of M199 (Thermo Fischer, #11 150 059) supplemented with 20 vol.‐ % FBS, 1 vol.‐ % P/S, 50 µg mL^−1^ endothelial cell growth supplement (ECGS, Corning, #356 006) and 25 µg mL^−1^ heparin (Sigma Aldrich, H3149) at 37 °C with 5% CO_2_ in a humidified incubator and used between passages 3 and 4. HUVECs were passaged at 80–90% confluency using 1× Trypsin/EDTA.

### Monocyte Isolation and Culture

Primary human monocytes were isolated from fresh human blood from healthy donors by a density gradient with Lymphoprep (StemCell Technologies, # 18 060) and SepMate‐50 tubes (StemCell Technologies, # 85 450) and negative isolation (EasySep Human Monocyte Isolation Kit, StemCell Technologies, # 19 359). Monocytes were cultured in ImmunoCult‐SF Macrophage Medium (StemCell, # 10 961) with 1% penicillin–streptomycin (ThermoFisher, # 15 140) and 50 ng mL^−1^ recombinant human GM‐CSF (Biolegend, # 572 903).

### iPSC‐HEP Differentiation and Culture

For deriving hepatocytes from human iPSCs we used a published protocol.^[^ ^43^ ^]^ We kept the cells for the complete differentiation at 37 °C in a 5% CO_2_ and 5% O_2_ atmosphere. Briefly, we cultured human iPSCs in mTeSR medium (STEMCELL technologies, # 85 850) including 10 µM Rock‐inhibitor (Tocris). With these we initiated hepatocyte differentiation on geltrex (Thermo Fisher, # A1413202) ‐coated 6‐well plates by exposing them to definitive endoderm (DE)‐Induction medium, consisting of RPMI1640, 2% B27 (Gibco), 1% PenStrep (Gibco), 100 ng mL^−1^ Activin A (Peprotech) and freshly added 50 ng mL^−1^ Wnt3 (RnD Systems), with 0.5 mM sodium butyrate (Sigma) for 5 days, and subsequently DE‐Induction medium without sodium butyrate for one additional day. Subsequently, we added medium consisting of KO‐DMEM (Gibco), 20% KO Serum (Gibco), 1% PenStrep (Gibco), 1% Glutamax (Gibco), 1% Non‐Essential Amino Acids (Lonza), 1% DMSO (PanBiotech) and 0.1 mM beta‐mercaptoethanol for 7 days to induce hepatic commitment. Next, we exposed cells for 7 days to HBM medium (Lonza, #CC‐3199) containing 20 ng mL^−1^ HGF (Peprotech), 20 ng mL^−1^ OSM (Miltenyi) and 1% PenStrep for hepatocyte commitment. We matured the cells for 7 days using complete HCM Bulletkit medium (Lonza, excluding EGF) with the additional supplementation of 5 nM T3 (Tocris), 100 nM sodium selenite (Sigma) and 100 µM CDCA (Sigma). After the maturation we utilized the cells for follow up assays.

### Cell Viability

*Live/dead*: to mark live and dead cells, Calcein‐AM (Biolegend, # 425 201) was diluted to a final concentration of 1 µM in DPBS (Thermo Fisher, # 14 190 144) together with Ethidium Homodimer‐I (Sigma Aldrich, #E1903) which was diluted to a final concentration of 4 µM. Cells or constructs were incubated for 15 min at 37 °C and either imaged on a Leica Stellaris 8 (for details, see imaging) or fluorescence signals were read using a Tecan Infinite 200Pro Microplate reader with 490/520 nm ex/em filter sets for Calcein/live cells and 530/640 ex/em filter for Eth1/dead cells. Raw reads were corrected by background fluorescence from a cell‐free control. For every experiment, a negative control was carried, created through incubation with a 0.1% Saponin solution dissolved in expansion medium while an untreated sample served as positive control. % Live cells were initially calculated by normalizing all signals to the positive control, while % Dead cells were calculated by normalizing all signals to the negative control. The viability expressed in % was then calculated as follows: %Via = %Live/(%Live + % Dead).

*Metabolic Activity*: Cellular metabolic activity was quantified using alamarBlue (Thermo Fisher, # DAL1100). The reagent was diluted in a 10:1 ratio in the respective cell growth medium and incubated for 4 h at 37 °C. The fluorescence was measured using a Tecan Infinite 200Pro with 560/590 ex/em filter sets. Raw reads were background subtracted and signals were normalized to an untreated sample that served as a positive control. Proliferation was quantified by normalizing values of metabolic activity after 5 days to day 1 (fold change) of each individual well and the Log 2 was calculated to derive population doublings.

### Histology

Both, 2D‐cultured cells and 3D objects were fixed using a 4% para‐formaldehyde (PFA) solution. The reaction was quenched using a 25 mM NH_4_Cl/DPBS solution. Staining was performed in Tris‐buffered saline (pH 8.2). For actin/nulei staining, samples were directly stained with the according dyes for 2 h, then washed three times with TBS. Antibody staining was performed using the following antibodies subsequent to permeabilization using 0.1% TBS‐Triton‐X100 solution and blocking using 1% BSA/5% normal goat/donkey serum diluted in TBS: CD31/PECAM‐1 (Thermo Fisher, #MA3100, HEC7 clone,), type I collagen (Abcam, #ab138492). Donkey‐anti‐rabbit (Thermo Fischer, #A‐21206), goat‐anti‐mouse (Thermo Fisher, #A‐11017) secondary antibodies were used for detection. The following dyes were used for visualization of actin cytoskeleton and nuclei: SYTOX Green (Thermo Fisher, #S7020), SYTOX Deep Red (Thermo Fisher, #S11381), Phalloidin‐Atto550 (Sigma Aldrich, #19 083).

Lightsheet samples were stained using a modified protocol and subsequent optical clearing: Samples were fixed in 4% PFA in PBS and followed by washing two times in PBS before permeabilization (0.5% *(v/v)* Triton X‐100 in PBS), and blocking 1% *(v/v)* BSA, 0.5% *(v/v)* Tween 20 in PBS) for 12 h each at 4 °C. Whole‐mount immunofluorescence staining was performed using alpaca polyclonal VHH anti‐human CD31 antibody, SYTOX Green nucleic acid stain (S7020, Invitrogen, Waltham, MA, USA) and Phalloidin Atto‐633 (68825‐10 nmol, Merck, Darmstadt, Germany) diluted in PermBlock solution. After staining, samples were washed three times in PBS‐T (0.1% *(v/v)* Tween 20 in PBS). The immunofluorescence‐stained spheroids were dehydrated through a series of increasing methanol concentrations (50%, 70%, 95%, >99.0%, >99.0% *(v/v)* methanol, each step for 60 min). The samples were subsequently optically cleared twice in a BABB solution (benzyl alcohol:benzyl benzoate solution, ration 1:2). The optically cleared spheroids were stored in BABB until imaging.

### Imaging

*Confocal imaging*: All images of cells in 2D and all 3D live/dead images were recorded on a Leica Stellaris 8 equipped with a 25× water immersion objective at an optical resolution of 1.2 × 1.2 × 2 µm voxel size. High resolution 3D zoom‐ins of the cell morphology were created with a 63× water immersion objective and an optical resolution of 0.12 × 0.12 × 1 µm voxel size. For live/dead images, a total volume of 1.6 × 1.6 × 0.4 mm was recorded and analyzed.

*Lightsheet imaging*: Immunofluorescence‐stained and optically cleared spheroids were optically sectioned using a Lightsheet 7 microscope (Zeiss, Oberkochen, Germany). Images stacks were acquired with a step size of 1.5 µm using Clr Plan‐Neofluor 20× detection optic at various magnifications. The digital 3D reconstruction of light sheet image stacks was conducted using the IMARIS Microscopy Image Analysis Software (Oxford Instruments plc, Abingdon, UK).

### Image Analysis

*Live/dead ratio (imaging‐based)*: Maximum projections of live and dead signals were thresholded and the binary mask was analyzed using the Analyze Particle function of ImageJ with regards of objects that are larger than 50 µm^2^ and a circularity of 0.25–1 to eliminate debris and noise signal. Signal intensity and thresholds were kept constant for all experiments.

*Cell Morphology analysis*: Maximum projections of the actin signal were created and after a median filter (radius = 1), the signal was thresholded and the binary mask was analyzed via the Analyze Particle function of ImageJ in order to obtain values of cell area and aspect ratio.

### CYP3A4 assay

The P450‐Glo CYP3A4 Assay (Luciferin IPA) (Promega, #V9001) was performed according to the manufacturer's instructions. In brief: The Luciferin‐IPA was diluted 1:1000 in iPSC‐HEP growth medium and incubated for 4 h at 37 °C. 25 µL of the supernatant was transferred into a white opaque 96well plate and 25 µL of the Luciferin Detection Reagent were added. Samples were incubated for 20 min before measuring luminescence using a Tecan Infinite 200Pro. Raw reads were background‐subtracted and individual samples were measured in duplicates. For normalization to cell count, the DNA content was quantified using the CyQUANT Cell proliferation assay kit (Thermo Fisher, #C7026) according to the manufacturer's instructions. In brief: Samples were frozen to support cell lysis and sample dis‐integration and thawed. The residual volume of the CYP3A4 assay was measured and a 2× working solution consisting of lysis buffer and GR dye was added and incubated for 20 min on a thermo‐mixer. 100 µL were transferred into a 96well plate and the fluorescence was measured with 485/530 ex/em filter sets using a Tecan Infinite 200Pro micro‐plate reader. Normalized CYP3A4 signals were obtained by relating raw luminescence reads to raw DNA signal reads representing cell count per condition.

### Printing

*Preparation of resin formulations*: Urethane‐methacrylate‐based prints were performed using the commercially available XoloOne‐375T formulation (Xolo GmbH). Synthetic hydrogel (PEGDA) prints were performed using the xoloPEGDA formulation kit (Xolo GmbH): Gelatin methacrylate formulations were based on X‐Pure GelMA 160P80 (Rousselot). The formulation contained 10% (wt. %) X‐Pure GelMA, 0.24 mg mL^−1^ DCPI5002, 0.5 M BisTris (pH 7.4). Cell‐containing formulations were additionally supplemented with 10% of 10× VAM Nutrient Mix (available from Xolo GmbH). A 10× cell suspension was prepared and 10% were added to the final print mix. The cell concentrations for viability testing were 0.5*10^6^ mL^−1^. For fibroblast‐driven ECM formation 10^6^ mL^−1^ were dispersed in the final print mix. Residual volume was filled with deionized water to achieve 100% total volume for each print mix.

*Printing*: Screening of printing conditions was performed with 10% GelMA, 0.24 mg mL^−1^ DCPI 5002 and 0.1–1 M BisTris dissolved in deionized water on a customized Xube setup equipped with two 375 nm Laser diodes and a projector (3840 × 2160 pixels). All other prints were performed on a commercially available 375 nm version Xube or Xube^2^ printer (Xolo GmbH). Printing conditions for cell‐containing constructs were 20 mJ at 1 mm min^−1^ print speed. For viability assessment in printed constructs, 8.5 mm diameter disks with 1.5 mm height were fabricated. For representation of printing capabilities, gyroids of varying porosity and unit cell size were used. The CAD files are available on request.

*Curing*: Curing was performed via a protocol adapted from previous reports.^[^ ^11^ ^]^ Following hydrogel printing of cell‐laden constructs, uncured resin was dissolved by heating to 37 °C and 2 consecutive washes with warm DPBS. Samples were further incubated for 5–10 min with 0.1% (wt./vol.) Lithium‐Phenyl‐2,4,6‐trimethylbenzoylphosphinat (LAP, Sigma Aldrich, #900 889) and photo‐cured using a Omnicure S2000 (Polytec GmbH) UV light source delivering 10 W cm^−^^2^ for 45 s. Cured print objects were briefly rinsed in DPBS and then either subjected to live/dead staining or culture in the respective growth medium.

### Rheology

Hydrogel discs of a final 8 mm diameter were printed and post‐cured, then equilibrated in PBS overnight. Rheological characterization was performed with a viscometer Anton‐Paar MC302 (Anton Paar, Graz, Austria) using an 8 mm parallel plate geometry. Measurements were performed using a strain rate of 0.1% at 1 Hz for 90 s. Averaged data from the last 50 s were used to calculate the storage and loss modulus for each sample. A minimum of three replicates were used per condition.

### Statistics & Data Presentation

Data are presented as mean values with standard deviation of at least 3 independent experiments, if not indicated otherwise. Dots indicate single measurements. Data plots were created using OriginPro 2020 (OriginLab Corporation). Significance was tested by Mann‐Whitney‐U test (two‐sided) with Bonferroni's correction for comparison of multiple groups (p^§^ = p × n). A value of *p* or *p* ^§^ < 0.05 was considered as statistically significant. Different significant levels are indicated as: # *p* < 0.1, * *p* < 0.05, ** *p* < 0.01, *** *p* < 0.001.

Chemical structures were created using ChemDraw 22.0.0. All figures and drawings were created using Inskape.

## Conflict of Interest

Niklas König is an employee of xolo GmbH and declares to have no competing financial or commercial interest that could have influenced the work reported in this study.

## Author Contributions

E.B. and A.B contributed equally to this work. A.P. and R.K.K. contributed equally as shared last authors on the work. E.B. conceptualized the study, designed and performed all main experiments, analyzed the data and wrote the manuscript. A.B. designed and performed the exploratory experiments and established the proof of concept of the approach, performed mechanical characterization and printing optimization tests, contributed to the writing of the introduction, manuscript editing, conceptual discussions, and supported the original consortium funding proposal with experiments and writing. M.R.K. performed and analyzed osmolality measurements and supported interpretation of associated experiments. J.W. and S.Q. cultured and differentiated iPSC‐HEPs under the supervision of M.R. with interpretation of associated results. R.S.K. isolated primary human monocytes. R.B. conducted light sheet imaging under the supervision of R.H. S.M. performed hydrogel formulation screening, supervised by M.M. and S.B. A.Ba. contributed to experiment discussions. M.F. facilitated project coordination and contributed to manuscript review. N.F.K. performed photophysical analysis of the DCPI and their interpretation. S.H. provided infrastructure and scientific input during project planning and manuscript preparation. A.P. supervised experimental work, provided infrastructure and mentoring and supported data interpretation. R.K.K. conceived and led the original consortium project, shaped the scientific direction throughout, coordinated cross‐group efforts, guided conceptual development, provided critical infrastructure and experimental strategy, and contributed to manuscript development and revisions. All authors reviewed the manuscript and approved its final version.

## Disclosure

N.F.K. is an employee of xolo GmbH and declares to have no competing financial or commercial interest that could have influenced the work reported in this study.

## Supporting information



Supporting Information

## Data Availability

The data that support the findings of this study are available from the corresponding author upon reasonable request.
