# Supplementary material for: Biocompatible Ink Optimization Enables Functional Volumetric Bioprinting With Xolography
Source: Adv Mater. 2025 Nov 29;38(6):e12058. doi: 10.1002/adma.202512058 (PMC12848640; doi:10.1002/adma.202512058)
Supplement: Supplementary file 1 — Supporting Information [file ADMA-38-e12058-s001.docx]

# Supplementary Data





**Supplementary Figure 1: (A)** Absorbance spectra of the DCPI at a concentration of 0.24 mg/mL at varying pH. **(B)** Difference spectra of the DCPI under UV irradiation at pH = 4, pH = 5 and pH = 6 relative to a dark reference control. The color code indicates individual time points ranging from 0 to 25 s of irradiation. **(C)** Thermal half life of the active merocyanine species derived from exponential decay fitting of 550 nm absorbance signal after switching off the UV light source.


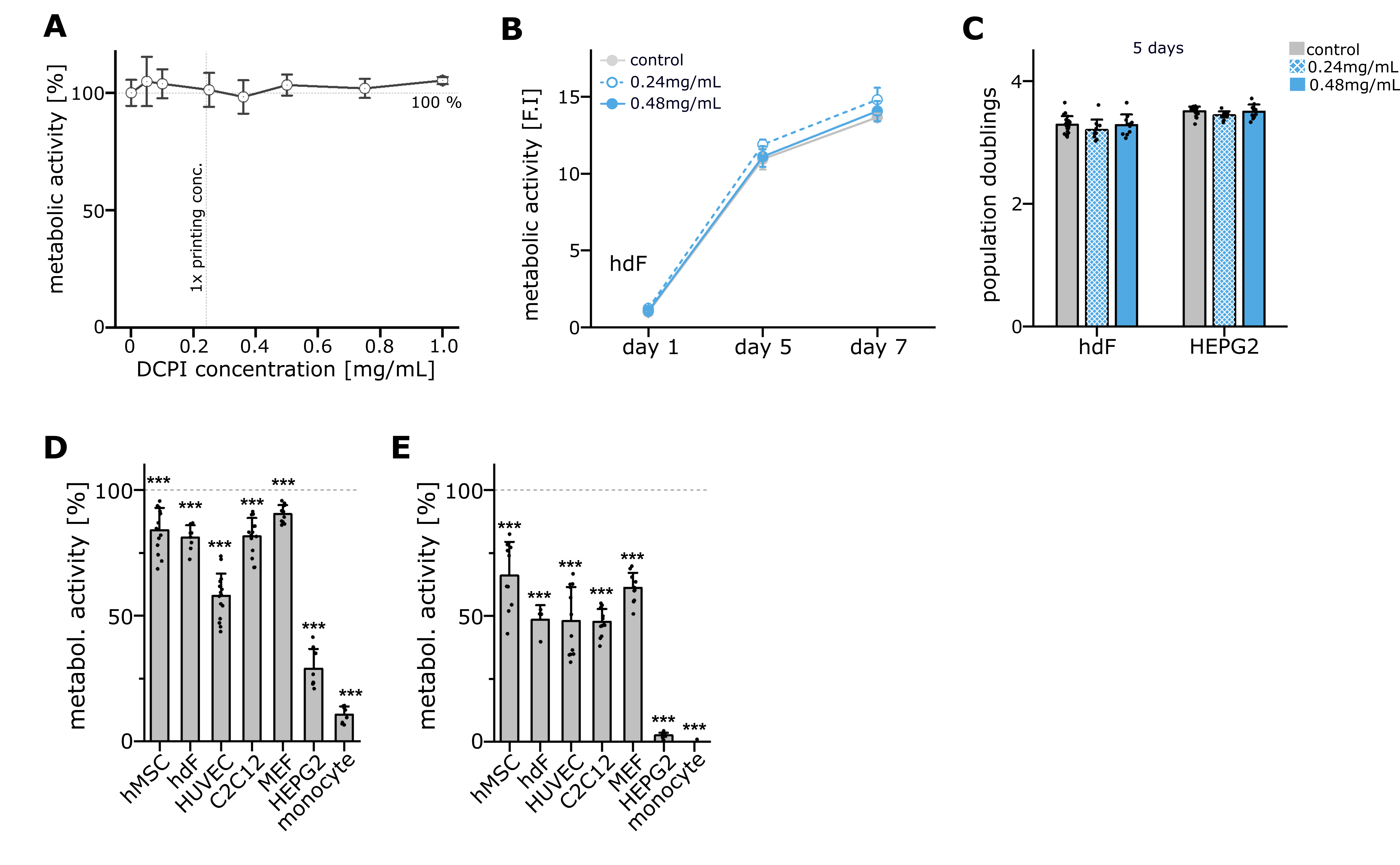


**Supplementary Figure 2: (A)** Metabolic activity of hdFs 24 h post treatment (normalized to untreated control cells) which were exposed to varying concentrations of the DCPI diluted in expansion medium. The gray dashed line represents the 1× concentration used for all other experiments. N > 5 of at least two independent experiments. **(B)** Metabolic activity of hdFs at day 1, 5 and 7 after treatment either with 0.24 mg/mL or 0.48 mg/mL of DCPI5002 diluted in expansion medium. Signals were normalized to the untreated control signal at day 1. N ≥ 10 of 2 independent experiments. **(C)** Proliferation rate of hdFs and HepG2 cells expressed as population doublings from day 1 to day 5. N ≥ 10 of 2 independent experiments **(D)** Metabolic activity 24 h post treatment (normalized to untreated control cells, gray dashed line) of cells which were exposed to 2.5 % TEOA diluted in growth medium. N ≥ 8 from at least two independent experiments. **(E)** Metabolic activity 24 h post treatment (normalized to untreated control cells, gray dashed line) of cells which were exposed to 2.5 % TEOA diluted in basal medium (FBS-free). N ≥ 8 from at least two independent experiments. Statistics via a two-sided Mann-Whitney-U test. A value of <0.05 was considered as statistically significant. Individual significance levels are indicated as follows: *: p<0.05, **: p<0.01, ***: p<0.001.





**Supplementary Figure 3: (A)** Amount of non-protonated TEOA molecules (expressed as % of all molecules) as function of pH according to the Henderson-Hasselbalch equation and a pK_a_ = 7.74. **(B)** **M**etabolic activity 24 h post treatment (normalized to untreated control cells) of cells which were exposed to growth medium titrated to pH = 9.5 and 25 mM HEPES to stabilize the pH. N ≥ 8 from at least two independent experiments. **(C)** Confocal images of endothelial cells either untreated or treated with pH 9.5 medium for 60 min. Cells were stained for actin (white) and nuclei (blue). Yellow arrows indicate lamellipodial protrusions. Scale bar 100 µm. **(D)** Medium osmolality measured by freezing point depression of a TEOA solution diluted in growth medium (1 % - 10 %) and PEG (MW = 400 g/mol) (5 % - 25 % wt./vol.). **(E)** Viability (% of live cells) of HEPG2 (light gray) and hdF (dark gray) cells shown as a function of PEG concentration (molecular weight: 400 g/mol) ranging from 5 to 25 % (wt./vol.%) after 60 min of exposure. N ≥ 8 from at least two independent experiments. **(F)** Metabolic activity (% untreated control) of HepG2 as a function of NH_4_Cl concentration (0 mM: blank, 125 mM: diagonally striped, 250 mM: diagonal cross) for controls (gray), alkaline pH (pH 9.5, yellow), osmotic pressure (15 % PEG, blue) or a combination of both (15 % PEG + pH 9.5, purple) after 24h of treatment for 60 minutes. TEOA (red) served as a reference for metabolic activity. N ≥ 10 from at least three independent experiments **(G)** Viability (% of live cells) of HEPG2 after 60 minutes of treatment either with 2.5 % TEOA (light red, dashed line) or 5 % TEOA (dark red) diluted in expansion medium at different pH conditions. N ≥10 of two independent experiments. Significance refers to control samples of the same pH condition. Right blue axis and dashed line indicate the amount of non-protonated TEOA molecules for the given pH condition. **(H)** Metabolic activity of hdFs at day 1, 5 and 7 after treatment either with 2.5 % or 5 % TEOA diluted in expansion medium. Signals were normalized to the untreated control signal at day 1. N ≥ 10 of 2 independent experiments. **(I)** Metabolic activity of HepG2 cells at day 1, 5 and 7 after treatment either with 2.5 % or 5 % TEOA diluted in expansion medium. Signals were normalized to the untreated control signal at day 1. N ≥ 10 of 2 independent experiments. **(J)** Proliferation rate of hdFs and HepG2 cells expressed as population doublings from day 1 to day 5. N ≥ 10 of 2 independent experiments. Statistics via a two-sided Mann-Whitney-U test. A value of <0.05 was considered as statistically significant. Individual significance levels are indicated as follows: *: p<0.05, **: p<0.01, ***: p<0.001.


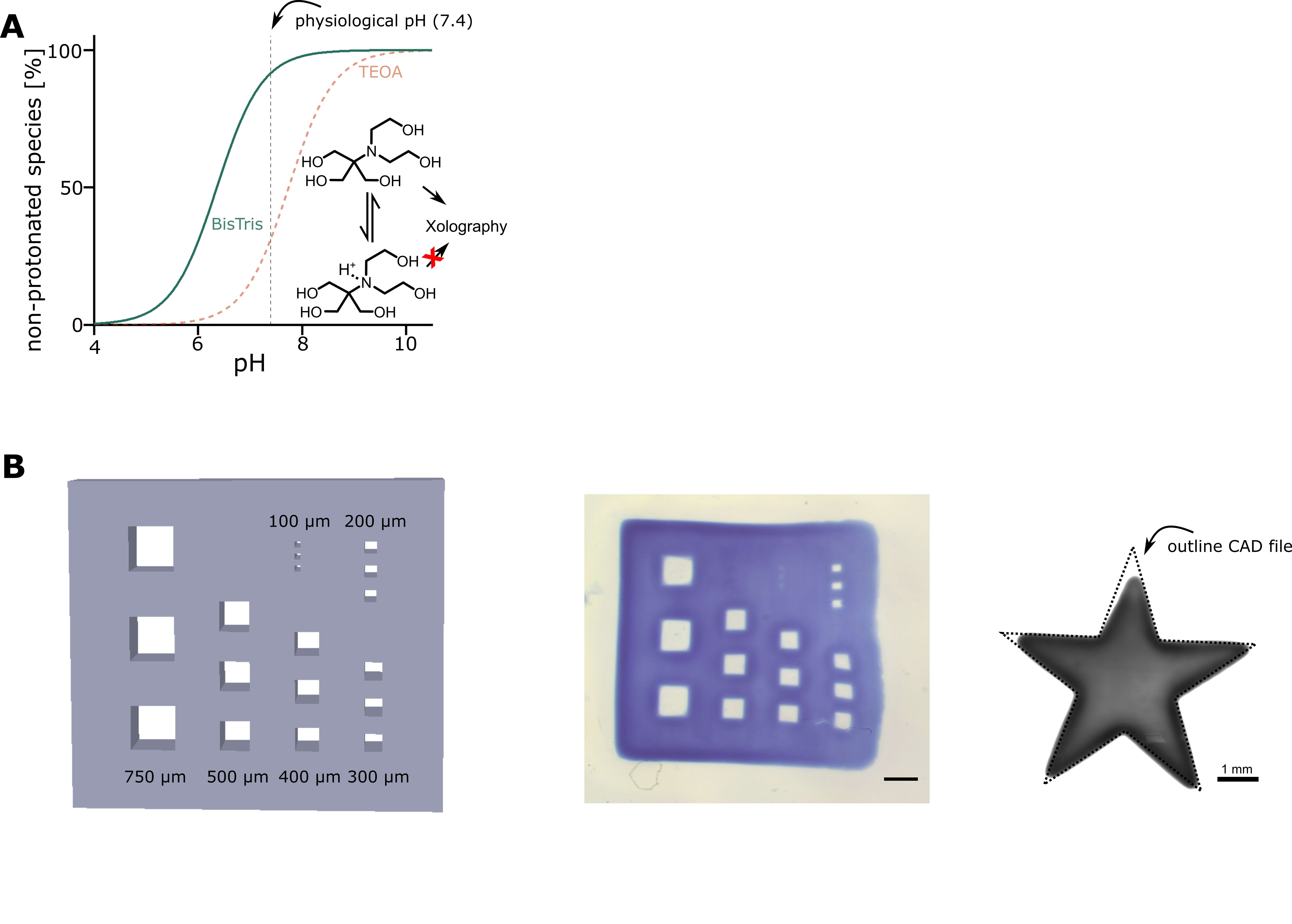


**Supplementary Figure 4: (A)** Amount of non-protonated TEOA and BisTris molecules (expressed as % of all molecules) as function of pH according to the Henderson-Hasselbalch equation. **(B)** Example prints using the biocompatible ink formulation (0.5 M BisTris, 10 % GelMA, 0.24 mg/mL DCPI5002) to print a feature resolution plate with rectangular holes of 750 – 100 µm side length. Test print was counter-stained using Coomassie Brilliant Blue for visualization of features. Right: 5-pointed star (phase contrast image) with overlaid CAD file outline (gray dashed line). Scale bar 1 mm.


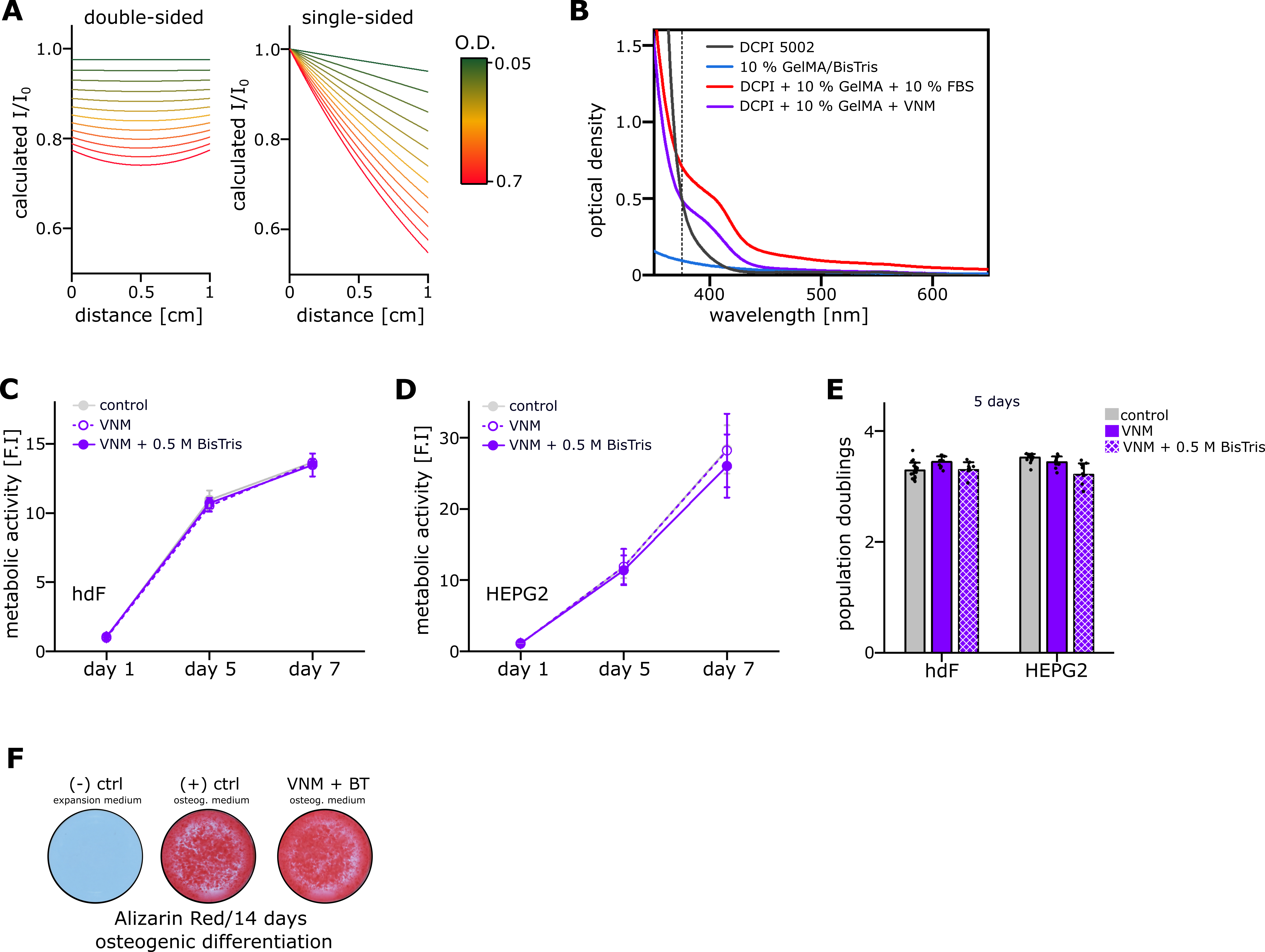


**Supplementary Figure 5: (A)** Light intensity distribution profiles calculated through Beer-Lambert law for different optical densities according to previous approaches^1^. A scattering coefficient of 7×10^-4^ was assumed. **(B)** Optical density of complete print mix formulations containing 10 % GelMA, 0.24 mg/mL DCPI 5002 and either 10 % FBS (mimicking standard growth media) or low-absorbing VMN. Optical densities for pure DCPI 5002 and 10 % GelMA are serving as a reference. **(C)** Metabolic activity of hdFs at day 1, 5 and 7 after treatment either with 1x VNM or 1x VNM + 0.5 M BisTris. Signals were normalized to the untreated control signal at day 1. N ≥ 10 of 2 independent experiments. **(D)** Metabolic activity of HepG2 cells at day 1, 5 and 7 after treatment either with 1x VNM or 1x VNM + 0.5 M BisTris. Signals were normalized to the untreated control signal at day 1. N ≥ 10 of 2 independent experiments. **(E)** Proliferation rate of hdFs and HepG2 cells expressed as population doublings from day 1 to day 5. N ≥ 10 of 2 independent experiments. **(F)** Alizarin Red staining of hMSC cultures which were differentiated in osteogenic differentiation medium for 14 days after a 60-minute exposure to VMN in 0.5M BisTris. An expansion medium sample served as a negative control.
